# Coordinated lignocellulolysis: topological analysis reveals coordinated lignocellulolysis in Klebsiella–Enterobacter-mediated rice straw degradation via surface delignification and cellulose crystallinity modulation

**DOI:** 10.1099/acmi.0.001097.v3

**Published:** 2026-05-12

**Authors:** Uvin Eksith Senadheera, Dikkumburage Jasintha Jayasanka, Choolaka Hewawasam, Dhanushka Udayanga, Yuya Takimoto, Nakayama Tadachika

**Affiliations:** 1 Department of Biosystems Technology, Faculty of Technology, University of Sri Jayewardenepura, Homagama 10200, Sri Lanka; 2 Faculty of Graduate Studies, University of Sri Jayewardenepura, Gangodawila, Nugegoda 10250, Sri Lanka; 3 Department of Civil and Environmental Technology, Faculty of Technology, University of Sri Jayewardenepura, Homagama 10200, Sri Lanka; 4 Department of Applied Sciences, Faculty of Humanities and Sciences, Sri Lanka Institute of Information Technology, SLIIT Malabe Campus, New Kandy Road, Malabe, 10115, Sri Lanka; 5 Department of Civil and Environmental Engineering, Nagaoka University of Technology, 1603-1 Kamitomioka, Nagaoka 940-2188, Niigata, Japan; 6 Extreme Energy-Density Research Institute, Nagaoka University of Technology, Nagaoka 940-2188, Niigata, Japan

**Keywords:** division-of-labour, *Enterobacter chuandaensis*, *Klebsiella variicola*, lignocellulolysis, rice straw degradation, rice straw endophyte

## Abstract

Surface-sterilized *Oryza sativa* AT362 straw was screened for endophytic ligninolytic and cellulolytic bacteria using colour unit reduction and Congo Red decolorization assays. Ligninolytic *Klebsiella variicola* AKL1104 and cellulolytic *Enterobacter chuandaensis* AKC1108, biocompatible, were inoculated at a 1 : 1 ratio and incubated in 1% (w/v) rice straw broth at 37 °C for 7 days. Topological changes due to degradation were conducted using scanning electron microscopy (SEM), X-ray diffraction (XRD), Fourier transform infrared spectroscopy (FTIR) and X-ray photoelectron spectroscopy (XPS) for 9 days. During *t*=9 d, SEM shows vascular tissue perforation with tunnels, indicating *K. variicola* mediated-delignification, allowing *E. chuandaensis* to sequentially degrade cellulose. XRD revealed a cellulose crystallinity decline from 30.08% on *t*=0 d to 11.76% on *t*=9 d. Electron microscopy and crystallite size calculations in *t*=6 d (6.81 nm) and *t*=9 d (90.16 nm) indicate self-assembly of cellulose fibrils. FTIR and XPS analysis indicated crystalline cellulose transformation to amorphous cellulose as the lateral order index dropped from 0.776±0.006 to 0.503±0.007, while surface lignin coverage was reduced from 5.01 to 2.20%, respectively.

## Graphical Abstract

*Klebsiella variicola* commences delignification, enabling *Enterobacter chuandaensis* to tunnel and decrystallize cellulose.

**Impact Statement**

This study reveals the division-of-labour-centric rice straw biodegradation by *Klebsiella variicola* AKL1104 and cellulolytic *Enterobacter chuandaensis* AKC1108 *K. variicola* AKL1104 and cellulolytic *E. chuandaensis* AKC1108. This study shows a coordinated lignocellulolysis of rice straw endophytes reported in degrading rice straw in a coordinated lignocellulolysis mechanism. The discovery of the endophytes with high efficiency in degrading their own lignocellulosic microenvironment gives a new insight into the literature, such that the utilization of local endophytic microbial population leads to enhanced lignocellulosic degradation instead of the introduction of extraneous specialized strains. We propose a division-of-labour pathway where *K. variicola* AKL1104 removes the surface lignin barrier first, while cellulolytic *E. chuandaensis* AKC1108 penetrates the exposed cellulosic matter, reducing the crystallinity index in a sequential attack. The generation of porous channels in the cellulosic material due to cellulolytic *E. chuandaensis* was observed in this study, which was previously unique and limited to cellulose degradation of *Bacillus* species. Furthermore, the study proposes a rigorously corrected surface-lignin-coverage equation for agricultural residues using XPS analysis with a mass-balanced model that supersedes the high-error $\frac{O}{C}$ approaches by integrating empirically derived stoichiometries and segment molar masses to offset lignin-spread carbon.

## Data Summary

16S rRNA sequences submitted in National Center for Biotechnology Information GenBank: AKL1104 (accession code: PP989911) and AKC1108 (accession code: PP989916). Supplementary files are available on Microbiology Society Figshare DOI: 10.6084/m9.figshare.29732339 [1].

## Introduction

Rice (*Oryza sativa* L.) is the primary staple food grain for a population of more than 3.5 billion people around the globe, especially in Asia, Latin America and some parts of Africa. Rice is an indispensable source of energy, fibre, vitamins, minerals and bioactive compounds [2]. It is the staple food in Sri Lanka. Due to the increased year-round demand, accelerated rice production causes the accumulation of rice straw. Rice straw is often disposed of by open-field burning. Rice straw open burning is a noteworthy source of air pollution. Rice straw burning releases a wide range of air pollutants such as carbon monoxide (CO), particulate matter, volatile organic compounds, polycyclic aromatic hydrocarbons and greenhouse gases such as nitrous oxide (N_2_O), carbon dioxide (CO_2_) and methane (CH_4_). These pollutants can lead to profound health problems and climate change. Furthermore, rice straw burning results in valuable nutrient loss of nitrogen (N), phosphorus (P), potassium (K) and sulphur (S) that could otherwise be recycled back into farm soil, reducing soil fertility.

Valorization of the rice straw biomass is a successful and sustainable strategy for the biotransformation of the rice straw into valuable products such as bioethanol and reducing sugars. However, the utilization of microbial consortia is challenging due to the recalcitrant nature of the lignocellulosic structure of rice straw [3]. According to Sahrawat and Garg [4], the major challenge for the progression of microbial consortia-mediated rice straw valorization is the depolymerization of lignin and the hemicellulosic barrier of rice straw. To overcome this issue, we utilize a sequential and combinatorial approach with a highly ligninolytic *Klebsiella variicola* AKL1104 endophyte and a highly cellulolytic *Enterobacter chuandaensis* AKC1108 endophyte isolated from rice straw inner tissues of *O. sativa* L. from the North Central province of Sri Lanka. To fully utilize this newly isolated consortium in an economically viable manner, it is essential to understand the mechanism of biodegradation induced by the two bacteria during rice straw degradation. We employ a two-stage approach to understand the mechanism of biodegradation. First, we investigated the topological and surface chemistry changes induced by the consortia during rice straw biodegradation and then the spectrometric approach to identify the secondary metabolites and intermediate products of biodegradation to unravel the pathways of biodegradation.

In this study, we present the topological and surface chemistry changes induced by the action of the bacterial consortium on rice straw degradation using spectrometric and topological analysis. The study’s objective is to fill the gap in the current understanding of topological and surface chemistry changes due to the biodegradation of rice straw substrates under the influence of endophytic *K. variicola* and *E. chuandaensis* and to introduce novel insights into surface lignin coverage calculations for an error-free estimation of surface lignin during rice straw biodegradation using X-ray photoelectron spectroscopy (XPS) while pointing out the flaws in already utilized equations in literature.

## Methods

### Rice straw sample collection

Rice straw residues of *O. sativa* L. cultivar AT362 were collected after the dry season of 2022 from paddy fields from *Tabuttegama* of *Anuradhapura* Mahaweli System H region (7.8293° N 81.4718° E), North Central Province, Sri Lanka. Ten kilograms of rice straw bundles was uniformly laid on surface-sterilized plastic sheets and misted with sterilized distilled water using an ordinary sterilized sprayer. The rice straw samples were placed in sterilized sampling bags and airtightly sealed for 48 h until they were transported to the laboratory. One kilogram of rice straw samples (randomly selected from different places of the initial sample) was surface-sterilized using 75% ethanol for 3 min, followed by vigorous shaking in 1.2% (w/v) NaOCl for 20 min. Rice straw samples with no visible surface fungal or bacterial infections were selected for further experiments [5]. The protocol described by Gyaneshwar *et al*. [6] is used for screening rice straw-inhabiting bacterial endophytes. The rice straw leaves are initially surface-sterilized with 70% ethanol for 5 min, followed by 0.2% HgCl_2_ treatment for 30 s. The secondary surface sterilization was done by submerging in a 50 ml 0.0.12% NaClO solution comprising 0.1% and 3% Na_2_CO_3_ and NaCl and 0.15% NaOH at 30 °C for 25 min in an orbital shaker at the rate of 200 r.p.m. The surface sterilization was completed by washing with 50 ml of Na_2_S_2_O_3_ to remove surface-adhered NaClO [7,8]. Under aseptic conditions, the surface-sterilized rice straw was separated into culms and leaf blades using sterilized forceps and scalpels (Hurek *et al*., 1994; Miche & Balandreau, 2001). The rice straw leaf blades and culms are surface subjected to secondary sterilization by dipping in 95% ethanol. Then the surface-sterilized rice straw is washed with sterilized distilled H_2_O 10 times with subsequent drying under laminar flow for 3 h at ambient temperature. The rice straw is examined for sterilization efficacy by rolling it on 0.1% tryptic soy agar (TSA) plates and cutting it into ≈2 cm pieces. 10.0 g of rice straw pieces are then crushed to create a homogenized paste with a sterile mortar and pestle in sterile 0.85% (w/v) physiological PBS (90.0 ml). The homogenate from rice straw pieces was centrifuged at 1,300 r.p.m. at room temperature for 10 min in aseptic conditions. The centrifuged supernatants are serially diluted up to 10^−5^, and each dilution is directly transferred into TSA to evaluate the total heterotrophic endophytic bacterial population. The endophytic bacterial isolates were selected based on distinct colony morphology, and the plating was repeated several times to obtain pure endophytic bacterial colonies. The pure colony isolates were grown in 7 ml Luria–Bertani (LB) broth comprising NaCl_2_ 10 g l^−1^, yeast extract 5 g l^−1^ and tryptone 10 g l^−1^ at 37 °C for 24 h. Storage and working stock of bacterial endophytes were made by 1.0 ml of culture in a 2 : 3 (v/v) sterile LB/glycerol mixture in sterile cryovials and stored at −80 °C and at 4 °C short term until further use.

#### Primary lignin-degrading ability screening of bacterial endophytes

Ten microlitres of culture aliquot pure bacterial strains was spot-inoculated into minimal salt media supplemented with lignin sulfonate (low sulfonate content) (MSM-L) to confirm the bacterial growth of endophytes with lignin sulfonate as the sole carbon source. The MSM-L composition is 2.0 g l^−1^ (NH_4_)_2_SO_4_, 0.5 g l^−1^ MgSO_4_, 1.0 g l^−1^ K_2_HPO_4_, 0.5 g l^−1^ NaCl, 5.0 g l^−1^ alkaline lignin, 20.0 g l^−1^ agar powder and 1.0 l H_2_O. A trace element solution of 1 ml^−1^ at pH 7.6 was introduced as described by Pfennig and Lippert [9], and the pH was adjusted to 7.6 using 1M NaOH. The aliquots were coated with a triangular glass rod (spread plate method), and the Petri plates were inverted and incubated at 37 °C for 7 days [10]. Endophytic bacterial isolates that were able to grow in MSM-L with lignin as the sole carbon source were subjected to further secondary screening to evaluate the degree of ligninolytic ability.

#### Secondary lignin-degrading ability screening

Secondary lignin-degrading ability screening was done to identify the endophytic bacterial isolate with the highest lignin-degrading potential. The lignin-degrading ability was evaluated spectrophotometrically for a period of 7 days. All the biodegradation evaluation assays were done in triplicate. The colour reduction due to biodegradation of lignin in broth culture was done according to the standard protocol of the Canadian Pulp and Paper Association [11]. 1.0 ml of overnight grown endophytic bacterial culture of inoculum size 105×10^3^ c.f.u. ml^−1^ on MSM-L (500 mg l^–1^) was inoculated into 250-ml flasks. Two millilitres of bacterial culture was collected by centrifugation at 12,000 r.p.m. for 5 min to remove the bacterial cells. 4.0 ml of Na_2_HPO_4_–NaH_2_PO_4_ buffer (pH 7.6) was introduced to the supernatant. The sample absorbance was measured at 465 nm against uninoculated MSM-L broth (control) using a spectrophotometer. Recorded absorbance values were converted into colour units (CU) using the following formula:


$$CU=500\times \frac{A_{2}}{A_{1}}\;\;\;\;\;\;\;\;\;\;\left(1\right)$$

$A_{1}$ represents the A456 of the 500-CU platinum-cobalt standard solution (Cole-Parmer, USA) (0.132), and *A*_2_ is the absorbance of the sample. The lignin biodegradation was evaluated as the ratio of the CU of the endophytic bacterial supernatant to the initial medium.

#### Primary cellulose-degrading ability screening of bacterial endophytes

Ten microlitres of culture aliquot pure bacterial strains was spot-inoculated into minimal salt media supplemented with carboxy methyl cellulose (CMC) (Na^+^ salt) to confirm the bacterial growth of endophytes with CMC as the sole carbon source. The MSM-CMC composition is 1 g l^−1^ KH_2_PO_4_, 0.5 g l^−1^ K_2_SO_4_, 0.5 g l^−1^ NaCl, 0.01 g l^−1^ FeSO_4_, 0.01 g l^−1^ MnSO_4_, 1 g l^−1^ NH_4_NO_3_, 10 g l^−1^ CMC, 20 g l^−1^ agar powder and 1.0 l H_2_O [12]. The plating, incubation and growth conditions are identical to those in the ‘Primary lignin-degrading ability screening of bacterial endophytes’ section.

#### Secondary cellulose-degrading ability screening

1.0 ml of overnight grown endophytic bacterial culture of inoculum size 105×10^3^ c.f.u. ml^−1^ on MSM-CMC centrifuged at 8,000 ***g*** for 15 min to collect the bacterial pellet. The collected bacterial pellets were washed with phosphate buffer (0.1 M; pH 6.5) to remove CMC interference with dye decolourization. The bacterial pellet is introduced into minimal saline agar medium with composition 1 g l^−1^ of Na_2_HPO_4_, 1 g l^−1^ of KH_2_PO_4_, 0.05 g l^−1^ of MgSO_4_, 3 g l^−1^ of NaCl, 0.05 g l^−1^ of CaCl_2_, 2 g l^−1^ of (NH_4_)_2_SO_4_ and in 1 l of distilled H_2_O containing 0.2% of glucose and 0.2% tryptone and supplemented with 5 mg l^−1^ of Congo Red (CR). The broth was incubated at 37 °C for 7 days. One millilitre of the bacterial suspension was collected and centrifuged at 10,000 ***g*** for 10 min to remove the bacterial mass. The obtained supernatant was evaluated for CR decolourization by UV spectrometry at $\lambda_{max}$ of 470 nm. Sterile broth cultures were used as controls. All the evaluations were conducted in triplicate.


$$CRdegradation\left(\%\right)=\frac{\left(A_{beforedegradation}-A_{afterdegradation}\right)}{A_{beforedegradation}}\times 100(2)$$

### Biocompatibility assessment

#### Biocompatibility assessment via spectrometric assay

The protocol by Sharma *et al*. [13] was used to evaluate the biocompatibility of selected ligninolytic and cellulolytic endophytic isolates. OD was evaluated for bacterial combinations at a 1 : 1 (ligninolytic/cellulolytic) ratio at OD_600nm_ after 1, 3, 5 and 7 days of incubation at 37 °C in modified succinate broth. The ratio of 1 : 1 was used to simplify the complexity of the biocompatibility test and to eliminate bias in OD reading due to a high growth rate of a strain and to keep all the conditions in the biocompatibility test equal, including the number of bacterial cells [13,14]. The initial total bacterial count was maintained at a concentration of 1×10^8^ c.f.u. ml^−1^ for single or combined inoculants. OD measurements were taken in triplicate. The top three dual endophytic consortia with the highest biocompatibility readings were used for the rice straw biodegradation assay.

### Rice straw degradation assay screening

A rice straw degradation assay was conducted for the bacterial combinations using the rice straw growth medium described by Gavande *et al*. [3]. The rice straw medium comprised 0.6 g l^−1^ of Na_2_HPO_4_, 3.0 g l^−1^ of KH_2_PO_4_, 5.0 g l^−1^ of NaCl, 1.0 g l^−1^ of NH_4_Cl, 0.5 g l^−1^ of MgSO_4_, 0.0015 g l^−1^ of CaCl_2_ and 1% (w/v) rice straw as the sole carbon source at pH 6.5. Rice straw was washed, dried at 90 °C for 8 h and milled. The milled rice straw is further refined by passing through a 60-mesh screen and is used in the growth medium [15]. One millilitre of each from dual endophytic consortium broth (inoculum size of 105×10^3^ c.f.u. ml^−1^) at a ratio of 1 : 1 was inoculated in rice straw medium at 37 °C for 7 days. The protocol for rice straw degradation evaluations was proposed by Du *et al*. [15] with minor modifications. Every 24 h, 10 ml of the culture was taken out and centrifuged at 10,000 r.p.m. for 10 min to remove the bacterial biomass. The obtained supernatant is washed with absolute 1M acetic–nitric solution for 20 min and, following washing with absolute ethanol for 20 min, to remove residual exopolysaccharides and non-lignocellulosic biomass in the supernatant. Rice straw media before bacterial degradation is considered a control (t=0)). The degradation (%) was evaluated using the following formula:


$$Ricestrawdegradation\left(\%\right)=\frac{W_{rs}^{after}}{W_{rs}^{before}}\times 100(3)$$

$W_{rs}^{after},W_{rs}^{before}$ are rice straw powder dry mass before and after degradation, respectively. The dual bacterial consortium with the highest rice straw degradation capacity was selected for further elucidation, spectroscopic and topological evaluation of rice straw degradation. Control experiments were conducted to determine the individual effects of single-strain systems on rice straw degradation based on the protocol described above.

### Molecular identification of the selected dual endophytic consortium

Dual endophytic consortium isolates were suspended separately in 500 µl ice-cold solution comprising lysozyme concentrations of 2 mg ml^−1^, followed by the addition of 10% SDS 50 µl and incubated at 37 °C for 5–10 min until the solution was clear and viscous. Then, the solution was transferred to a new tube, followed by adding 550 µl of phenol, which is freshly equilibrated with an equal volume of 0.3 M NaOCl. The contents of the mixture are mixed gently and subject to centrifugation at 15,000 ***g****,* 4 °C for 15 min. The top layer of the mixture was transferred to a new Eppendorf tube, and the previous step was repeated following another transfer of the top layer to a new Eppendorf tube. One-tenth of the volume of 3M naoi was added to the Eppendorf tube with two volumes of 100% ethanol. The solution was mixed by inverting. The sample was cooled for 5 min at −80 °C and subjected to centrifugation at 15,000 ***g*** for 15 min at 4 °C. The supernatant was discarded, and the pellet was vacuum-dried and resuspended with 50 µl of water. V3 and V4 regions of the 16srRNA gene were amplified. 336F (GTACTCCTACGGGAGGCAGCA) and 806R (GTGGACTACHVGGGTWTCTAAT) forward and reverse primers were used with the extracted DNA sample as the template. Twenty-five microlitre reaction mixture comprised of 5×PCR buffer (5 µl), 2 mM dNTPs (2.5 µl), 25 mM MgCl_2_ (2.5 µl), 5 µM 336F forward primer (1 µl), 5 µM 806R reverse primer (1 µl), template DNA (5 µl) or nuclease-free water (5 µl; negative control), GoTaq Flexy DNA polymerase (0.25 µl) and nuclease-free water (7.75 µl). PCR was conducted using a Bio-Rad^™^ PCR Thermal Cycler. The cycling specifications are denaturation at 95 °C for 30 s, annealing at 56 °C for 30 s, extension at 72 °C for 30 s and final extension at 72 °C for 10 min. The obtained amplicons in the PCR assay were confirmed by 0.8% agarose gel electrophoresis, following staining with SyberSafe dye. The amplified products were subjected to Sanger sequencing using 336F (GTACTCCTACGGGAGGCAGCA) and 806R (GTGGACTACHVGGGTWTCTAAT) forward and reverse primers. The sequence homology was compared utilising Basic Local Alignment Search Tool (blast) (https://www.ncbi.nlm.nih.gov/blast/) against the National Center for Biotechnology Information (NCBI) database. The evolutionary distances were computed using the maximum composite likelihood method [16] and are in the units of the number of base substitutions per site. Evolutionary analyses were conducted in mega v12 utilizing up to 4 parallel computing threads and 1,000 bootstrap replicates [17].

### Growth curve determination of each selected endophytic isolate

To determine the growth pattern of the dual endophytic consortium, 1.0 ml of grown endophytic bacterial culture of an inoculum size of 105×10^3^ c.f.u. ml^−1^ were transferred separately into MSM-L and MSM-CMC broth (500 mg l^–1^) and incubated at 37 °C for 7 days. Every 24 h, 1.0 ml of the sample was withdrawn from the broth, and the growth curve was anticipated by UV absorbance increase monitoring at 620 nm for ligninolytic isolate and 600 nm for cellulolytic isolate, respectively [17]. Cultures prior to bacterial growth were used as controls (*t* = 0). Readings were taken in triplicate.

### Biochemical characterization of a dual endophytic consortium

The approach used by Raj *et al*. [18] was used for the physiological and biochemical characterization of the dual endophytic consortium.

### Topological changes analysis of rice straw biodegradation by the dual endophytic consortium

The bacterial combination (ratio of 1 : 1) comprising one ligninolytic endophyte and one cellulolytic endophyte with the highest rice straw degradation potential (‘Rice straw degradation assay screening’ section) was used for topological changes analysis of rice straw biodegradation of the endophytic consortium. Every 3 days, 1.0 g of rice straw sample was obtained by subjecting to centrifugation at 10,000 r.p.m. from incubated rice straw medium (2.4) with a dual endophytic consortium (1 : 1) and washed with distilled H_2_O and dried at 60 °C for 24 h (*t* = 0 d, *t* = 3 d, *t* = 6 d, *t* = 9 d). To remove any residual moisture left, the dried rice straw sample is freeze-dried for 24 h. The obtained sample is used for scanning electron microscopy (SEM), X-ray diffraction (XRD), FTIR (Fourier-transform infrared spectroscopy), XPS and atomic force microscopy for the evaluation of topological and conformational changes in rice straw substrate due to endophyte-mediated biodegradation.

#### SEM analysis

SEM analysis was conducted as described in Dar *et al*. [19].

#### XRD analysis

Diffraction specifications are in the grade range of 10°–50° with a step size of 0.02°. A radiation source is Cu-1.54 Å radiation at 30 kV with 10 mA, with a single continuous scan mode for observation of rice straw biomass (reference). The approach proposed by Segal *et al*. [20] is used to calculate the cellulose crystallinity index (*CrI*). The pragmatic equation is as follows:


$$CrI\left(\%\right)=\left[\frac{\left(I_{002}-I_{am}\right)}{I_{002}}\right]\times 100(4)$$

$I_{002}$ and $I_{am}$ are the corresponding region intensities of cellulose in rice straw substrates, where $I_{002}$ and $I_{am}$ are peak heights at 2*θ* = 22.5 (corresponds to the crystalline cellulose region) and 18.6 (corresponds to the amorphous cellulose region), respectively.

Crystalline size (CS) is calculated by the Scherer equation [21] as follows:


$$CS=\frac{k\lambda }{\beta cos\theta }\;\;\;\;\;\;\;\left(5\right)$$

k is the dimensionless shape factor (0.94), *β* = the full width at half maximum intensity of the peak in radians, *θ* = Bragg’s angle and λ= X-ray ionization wavelength of 0.1542 Å. All XRD readings were taken in triplicate.

#### FTIR analysis

Ten milligrams of freeze-dried rice straw substrate was used for the KBr method. The sample was thoroughly mixed with 1,000 mg of spectroscopic-KBr in an agate mortar. The mixture is pressurized (up to 10,000 psi) to obtain a fairly transparent thin pallet. The FTIR spectra were obtained in the wavelength range of 4,000 cm^−1^ to 400 cm^−1^ at 4 cm^−1^ spectral resolution with 32 scan accumulations. The IR CrI was calculated as described by Satyamurthy *et al*. [22]. The S(syringyl)G(guaiacol) ratio, lateral order index (LOI), total crystallinity index (TCI) and hydrogen-bonding index (HBI) were calculated as described by Koutsianitis *et al*. [23]. All the readings were taken in triplicate.

#### XPS analysis of rice straw degradation

Rice straw samples were etched in Ar for 30 s prior to XPS analysis to eliminate the effect of any residual impurities. The XPS was done using Thermo Scientific™ ESCALAB Xi^+^ X-ray photoelectron spectrometer with monochromated Al K*α*-X-rays with 10 eV Auger electron source. The survey obtained high-resolution spectra for C_1s_, O_1s_ and N_1s_ regions from 0 to 1350 eV with a spot size of 900 µm. Scans were performed in an ultra-high vacuum Mu-chamber with ≤5×10⁻¹⁰ mbar with sample preparation at 7×10⁻⁹ mbar. To improve sample representativeness and due to heterogeneity of the sample, two independent surveys (survey A and B) were done on two different locations (*n* = 2) within the sample, and the average was taken for the final narrow and broad surveys [24]. The C_1s_, O_1s_ and N_1s_ spectra were deconvoluted into distinct peaks. Peak fitting was performed using Gaussian functions with background subtraction. Surface lignin coverage (SLC) and surface elemental conformation of degraded rice straw are calculated from OC ratios according to the following equation [25]:


SLC%=OCsample-OClignin-OCcellulose×100(6)

OCsampleare the OC ratio of the rice straw sample, OClignin is theOC ratio of pure lignin (0.33) and OCcellulose is the OC ratio of pure cellulose (0.83) [25]. The peak analysis was done by means of rice straw decomposition changes of peaks to fit into a Gaussian function. The ratio of oxygenated to unoxygenated carbonCoxygenatedCunoxygenated was obtained from the equation below.


CoxygenatedCunoxygenated=C2+C3C1(7)

The acid-base balance was calculated using the following equation:


Acid-Basebalance=(C2)(C1)(8)

### Statistical analysis

Biocompatibility and FTIR analysis were performed by two-factor ANOVA and one-way ANOVA with Tukey’s (HSD) Honestly Significant Difference post hoc analysis (*p* < 0.05), respectively. XRD analyses were performed with a first-order decay model with 2,000-fold bootstrap confidence intervals (CIs), followed by Pearson and Spearman correlations for CrI and CS (significant at *p* < 0.05). XPS analysis was performed by trend analysis of the single-spectrum time-series (0 days, 3 days, 6 days, 9 days; *n* = 4) with 2,000-fold bootstrap 95% CI and exact Mann–Kendall tests (*maximum S* = 6; *p* < 0.05). *Python v3.9* and *Microsoft Excel v2019* were used for statistical analysis.

## Results and discussion

### Ligninolytic and cellulolytic screening

#### Screening and biocompatibility of endophytic isolates

**Fig. 1.**
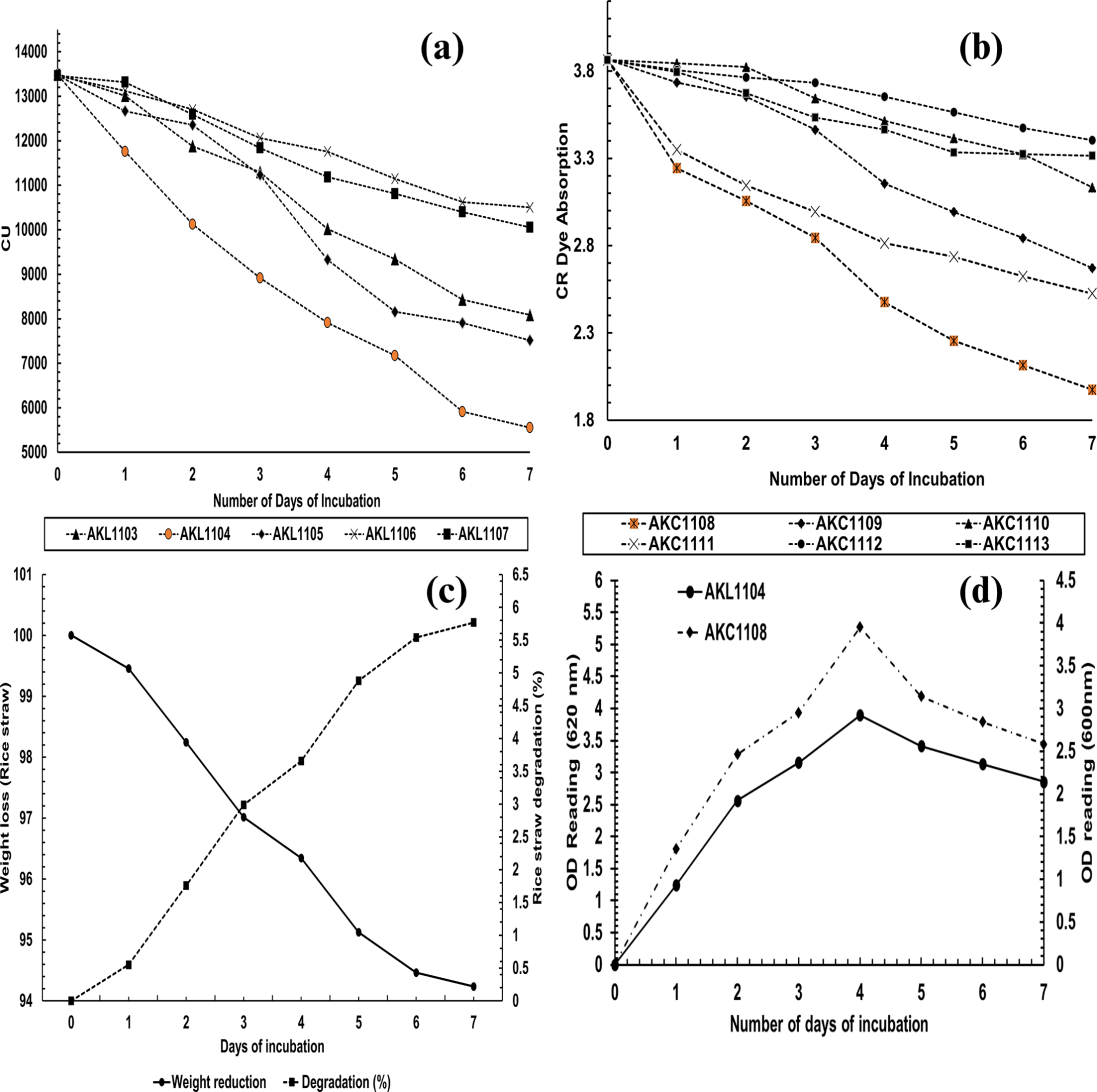
**(a**) The lignin decolorization potential of the screened ligninolytic bacterial endophytes (the highest isolate highlighted in orange). (**b**) CR decolorization potential of the screened cellulolytic bacterial endophytes (the highest isolate highlighted in orange). (**c**) Rice straw degradation pattern of the selected dual consortium (1 : 1). (**d**) Growth curve of the selected ligninolytic and cellulolytic isolates.

**Fig. 2.**
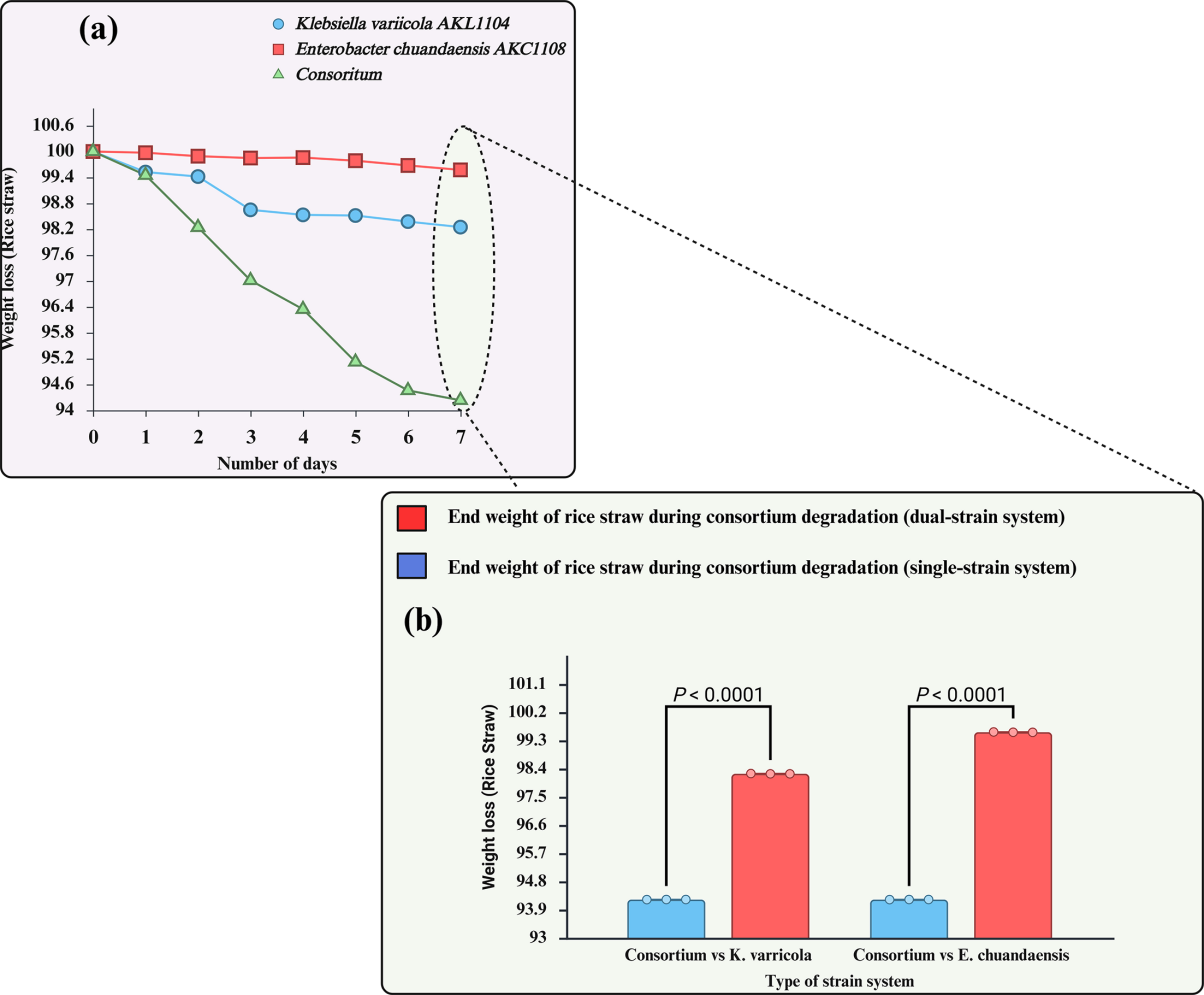
**(a**) The rice straw degradation (weight loss) analysis of dual-strain and single-strain systems. (**b**) Direct comparison of the end weight of rice straw analysis of dual-strain and single-strain degradation. Two-way ANOVA with the Bonferroni multiple-comparisons test confirms the statistical synergistic effectiveness of dual-strain systems compared to single-strain systems.

A total of 41 bacterial endophytic isolates were plated from the inner tissues of rice straw from the *Tabuttegama* area. Six bacterial isolates were able to grow in MSM-CMC (500 mg l^−1^), while five bacterial isolates were able to grow in MSM-L (500 mg l^−1^), indicating the presence of ligninolytic and cellulolytic endophytic communities inside rice straw tissues, confirming earlier studies by Das *et al*. [26]. Fig. 1(a) shows the ligninolytic degradation potential for the ligninolytic endophytes. The highest lignin degradation potential (58.818±0.008%) was recorded in isolate AKL1104, while AKC1108 showed the highest cellulose degradation potential (48.887±0.025%) (Fig. 1b). Furthermore, based on optical density readings, the highest biocompatibility between the given cellulolytic and ligninolytic endophytic isolates was observed between AKL1104 and AKC1108, which showed the highest ligninolytic and cellulolytic potential. The optical density readings (quantitative assessment) of biocompatibility results are shown in Table 1 for selected endophytes. AKL1104+AKC1108 showed the highest rice straw biodegradation efficiency with the highest mean OD increase of 0.101 after 7 days and the mean rice straw degradation percentage of 5.767% (wt.%) (Fig. 1c). Therefore, AKL1104+AKC1108 were selected as the preferred dual consortium for further topological and surface chemistry characterization of rice straw degradation for elucidation of the mechanism of degradation. The growth curve of both isolates was similar to each other with maximum growth indicated in MSM-L (500 mg l^−1^) and MSM-CMC (500 mg l^−1^) on the 4 days of incubation (Fig. 1d). During the utilization of single strains for rice straw degradation, a mean degradation of 1.75% and 0.42% was recorded for AKL1104 and AKC1108 single-strain systems, respectively (Fig. 2a). It is important to note that the rice straw biodegradation is statistically significant during the utilization of consortium (*p* < 0.0001) compared to the single-strain degradation (Fig. 2b).

**Table 1.** Dual combination biocompatibility assay for selected endophytes (*n* = 3)*

Bacterial isolate combination (dual)	No. of days of incubation (OD _600nm_ readings; mean±sd)			
	1 day	3 days	5 days	7 days
AKL1103+AKC1108	0.116±0.006^gD^	0.163±0.001^hC^	0.184± 0.0005^gB^	0.195±0.002^gA^
AKL1103+AKC1109	0.151±0.010^fC^	0.172± 0.001^gB^	0.184±0.002^gA^	0.193±0.001^gA^
AKL1103+AKC1111	0.154±0.002^fD^	0.164±0.002^hC^	0.174±0.001^hB^	0.183±0.002^gA^
AKL1104+AKC1108	0.667±0.0005^aD^	0.982±0.002^aC^	1.225±0.001^aB^	1.636±0.001^aA^
AKL1104+AKC1109	0.423± 0.001^cD^	0.613±0.001^dC^	0.853± 0.002^dB^	1.156± 0.057^dA^
AKL1104+AKC1111	0.634±0.0005^bD^	0.933±0.001^bC^	1.144±0.001^bB^	1.432±0.001^bA^
AKL1105+AKC1108	0.624±0.001^bD^	0.912± 0.001^cC^	1.032±0.0005^cB^	1.253±0.001^cA^
AKL1105+AKC1109	0.374±0.0005^dD^	0.533±0.001^eC^	0.721±0.001^fB^	0.943±0.001^fA^
AKL1105+AKC1111	0.332±0.001^eD^	0.434±0.002^fC^	0.822±0.001^eB^	1.043±0.001^eA^

*Values are presented as mean±sd. Biocompatibility screening revealed a dominant consortium (dual combinations) effect (two-factor ANOVA: *p* < 0.001; partial *η*2 = 0.99 with a significant consortium ×time interaction (*p* < 0.001). Day-wise Tukey HSD showed that AKL1104+AKC1108 had the highest biocompatibility (*p* < 0.001). Lowercase letters indicate nine combinations within each incubation day, and the same lowercase letters indicate not significantly different (Tukey’s HSD test, *α* = 0.05) within incubation time-point. Uppercase letters indicate four time-points (1 day, 3 days, 5 days and 7 days) within the same combination, and the same uppercase letters indicate no significant change over time (Tukey’s HSD test, *α* = 0.05).

#### Molecular and biochemical identification of the ligninolytic AKL1104 and cellulolytic AKC1108 dual consortium

**Fig. 3.**
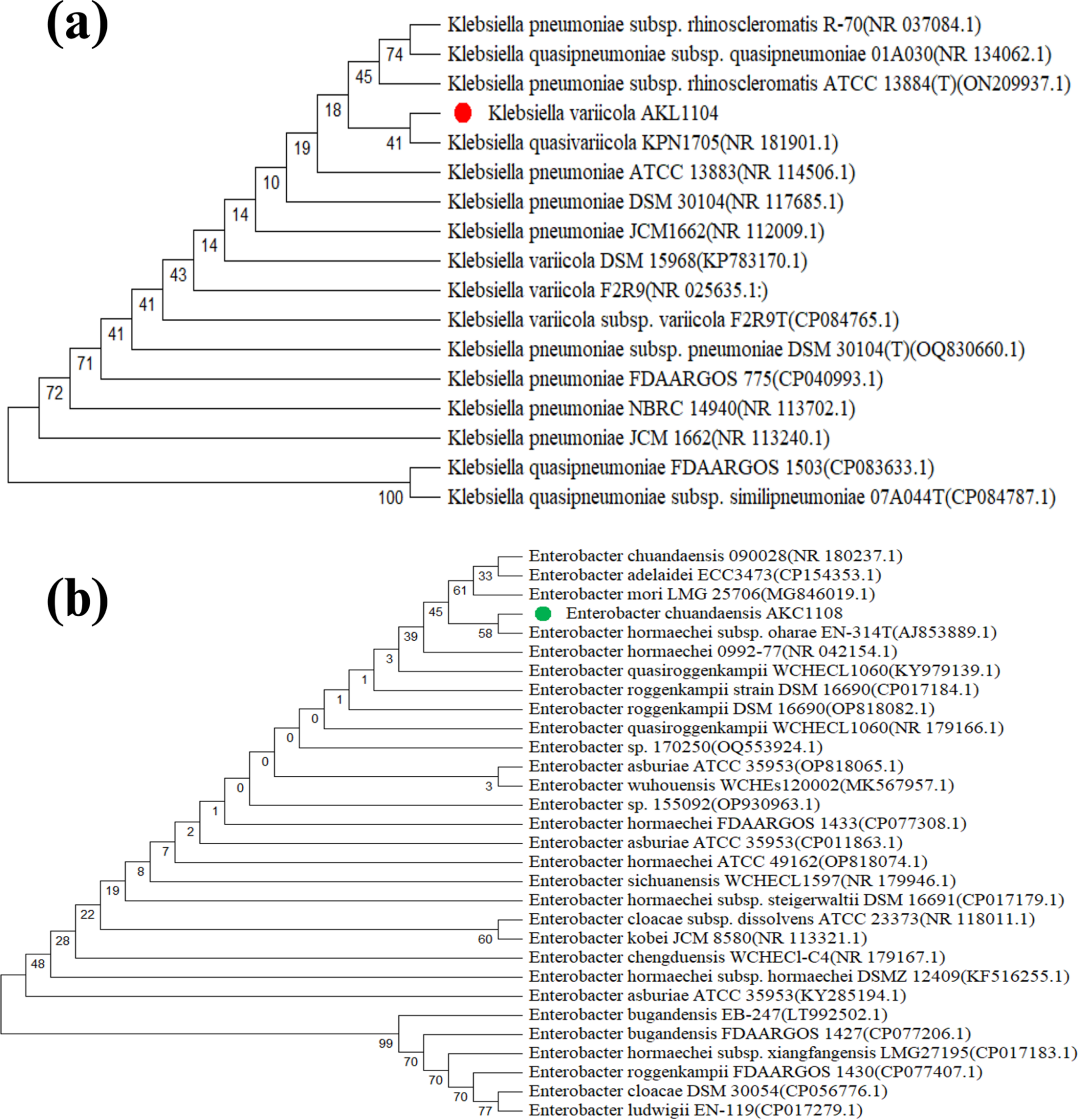
**(a**) The phylogenetic relationship of *K. variicola* AKL1104. The percentage of replicate trees in which the associated taxa clustered together in the bootstrap test (1,000 replicates) is shown below the branches [77]. The pairwise deletion option was applied to all ambiguous positions for each sequence pair, resulting in a final data set comprising 465 positions. (**b**) The phylogenetic relationship of *E. chuandaensis* AKC1108. The percentage of replicate trees in which the associated taxa clustered together in the bootstrap test (1,000 replicates) is shown below the branches. The analytical procedure encompassed 30 nt sequences. The pairwise deletion option was applied to all ambiguous positions for each sequence pair, resulting in a final data set comprising 450 positions.

The 16S rDNA sequence was deposited in NCBI GenBank and obtained the accession ID of the blast results as PP989911 and PP989916 for ligninolytic AKL1104 and cellulolytic AKC1108, respectively. The DNA sequence of AKL1104 was 99.7% homologous with blast hits of *Klebsiella* spp. Nevertheless, the biochemical characteristics of the isolate determined that AKL1104 is closely related to *K. variicola* (Table 2) [27]. There are records of cellulolytic nitrogen-fixing *Klebsiella* isolates characterized as paddy soil inhabitants [28]. However, the existing scientific literature lacks evidence of records on the identification and characterization of ligninolytic *K. variicola* as a rice straw endophyte. Likewise, the AKC1108 isolate was 99.5% identical to blast hits of *Enterobacter* spp. The biochemical characterization of the isolate indicates that it is closely related to *E. chuandaensis* (Table 2). There are no current records of isolation of cellulolytic *E. chuandaensis* as a rice straw endophyte. *Enterobacter* species has been identified as rice straw endophytes previously [29]. However, the existing scientific literature lacks evidence of records on the isolation and characterization of cellulolytic *E. chuandaensis* as a rice straw endophyte. The *E* values of both blast searches were zero. Phylogenetic tree showing the evolutionary relationship of both isolates in Fig. 3(a) (*K. variicola* AKL1104) and Fig. 3(b) (*E*. AKC1108), respectively. Reports by Chang *et al*. [30] and Jiménez *et al*. [31] strongly support the synergistic rice straw biodegradation by *K. variicola Enterobacter* species.

**Table 2.** Biochemical characterization of *K. variicola* AKL1104 and *E. chuandaensis* AKC1108

Physiological and biochemical test	Result	
	*K. variicola* AKL1104	*E. chuandaensis* AKC1108
Shape	Rod-shaped (single, in pairs or short chains)	Rod-shaped (elongated and cylindrical)
Gram reaction	-−	-−
Cellular length and diameter	0.3–1 µm-diameter, 0.6–6 µm-length	0.6–1.0 µm-diameter, 2–3 µm-length
Motility	−	+
Growth on nutrient agar (NA)	No growth	No growth
Growth in the air	+	+
Anaerobic growth	+ (facultative anaerobe)	+ (facultative anaerobe)
Growth at 50 °C	-−	-−
Growth in 10% NaCl	−	−
Catalase	+	+
Oxidase	-−	-−
Oxidative/fermentative	Oxidative and fermentative	Fermentative
Acid production	−	−
Cellobiose	+	+ (fast)
Fructose	+ (fast)	+
Lactose	+ (slow)	+
Mannose	+ (fast)	+
Raffinose	+ (slow)	+ (fast)
Xylose	+ (fast)	+
Casein hydrolysis	+	+
Starch hydrolysis	+	+ (slow)
Citrate utilization	-−	+ (slow)
H_2_S production	-−	−
Indole test	-−	-−
Urease	− (slow)	+ (slow)
Nitrate reduction	+	+ (fast)

The *K. variicola* AKL1104 and *E. chuandaensis* AKC1108 consortium showed similar biochemical characteristics. We suspect that this might be due to the mutual evolution of similar microenvironments of rice straw tissue and physiological pressure that forced the isolates to consume similar kinds of nutrients and develop metabolic pathways (Table 2).

#### Topological changes analysis of rice straw biodegradation by the dual endophytic consortium

##### SEM analysis – rice straw degradation

**Fig. 4.**
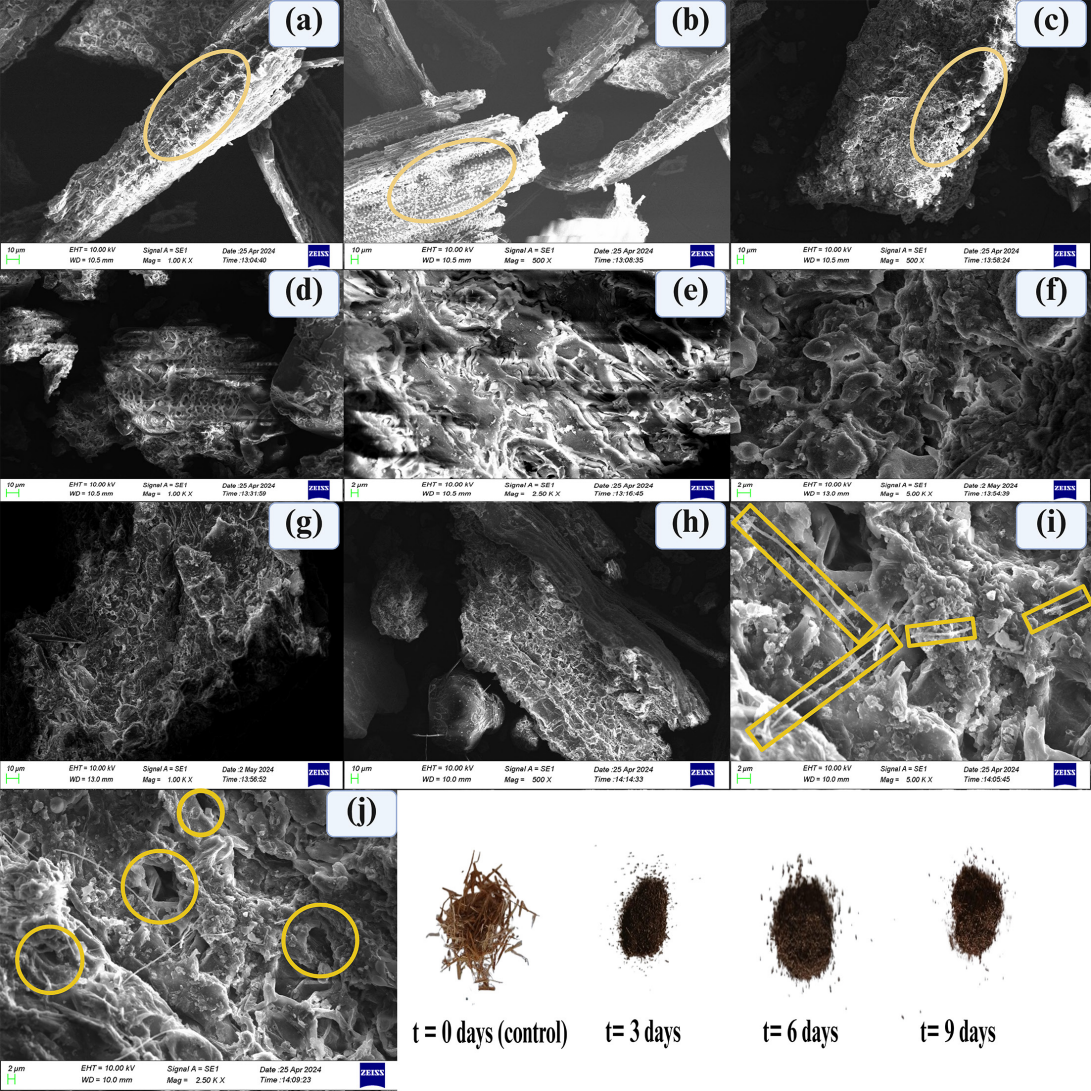
**(a**) The surface morphology of undegraded rice straw powder *t* = 0 d (control) with epidermal cell remnants (Fig. 3)**. (b**) The protuberances and trichomes on the surface of rice straw powder. (**c**) Destruction of protuberances and trichome structures (*t* = 3 days). (d) A wave-like distinctive degradation pattern visible on the surface of rice straw powder. (**e**) Enlarged view of the wave-like degradation patterns. We suspect this is due to the laccase enzyme attack of *K. variicola* AKL1104. (**f**) Irregular morphologies due to delignification and destruction of cell shapes (enlarged image). (**g**) Zoomed out image of Fig. 3). **(i**) The vascular tissue exposure due to degradation. (**j**) The presence of scattered microfibrils on the powder surface is indicated by red coloured rectangles (mean length: 10.87±4.9 µm; mean diameter: 0.78±0.19 µm). The presence of tunnels of varying dimensions deep into the cellulosic matter. Even though the reports by Blanchette [36] provide room for the potential tunnelling effect of *E. chuandaensis,* this is one of the strong evidence of *E. chuandaensis* contributing to a CrI reduction through the microscopic tunnelling effect. Furthermore, the SEM micrographs show clear evidence of microscopic tunnelling effect on CMC surface (initiation of tunnelling via concentric lesions) and rice straw surface, suspected to be due to cellulolytic action of *E. chuandaensis* AKC1108 (refer to supplementary materials).

In t=0 d, the cell wall composition of powdered rice straw can be clearly seen, including epidermis, parenchyma adhered to the bundle surface and vascular bundles [31]. The abaxial and adaxial sides of the rice straw blades comprise many protuberances and trichomes, as shown in Fig. 4(a, b). The areas with a smaller number of protuberances and trichomes are subjected to degradation more effectively due to the enhanced enzyme access to hemicellulose and cellulose without the shielding effect of lignin. In day *t*=3 d, of bacterial degradation of rice straw powder, the protuberances and trichome structures are being completely altered (Fig. 4c). Localized degradation in the form of lesions is visible as wavy patches (Fig. 4c). The rice straw surface revealed a rough surface with cracks and was subjected to delamination with visible pores and crevices. Similar degradation morphologies are reported by Fu *et al*. [32]. The cracks and crevices formed are caused by, or might be due to, ligninolytic action of *K. variicola* AKL1104. We suspect that this action enhances the cellulase accessibility and promotes the subsequent cellulose degradation in rice straw.

During the *t* = 6 d of degradation, the later stage of rice straw biodegradation is prominent with prolonged morphological changes, deeper and angular grooves of degradation and increased pore sizes. These observations are consistent with the study conducted by Liu *et al*. [33]. A similar pattern of surface degradation was observed by Ningthoujam *et al*. [34], where 4% NaOH was used for chemical pretreatment of rice straw. Upon closer examination, a localized wave-like degradation is due to fibre abrasion and fibre splitting with loosening of the middle lamella, primary and secondary cell walls of parenchymal cells (Fig. 4d, e). Dring *t* = 6 d of rice straw biodegradation, in some regions, a more prominent degradation pattern is visible on the surface. Fibre bundles are highly loosened, while the surface area is increased. The severe delignification has exposed the interior tissues and cellulosic matter (Fig. 4f, g). On the *t* = 9 d of biodegradation, the vascular tissue of rice straw is entirely exposed, indicating severe degradation. This is consistent with the observations of Xia *et al*. [35]. Cellulosic fibre destruction has caused the removal and release of cell shapes from rice straw surface (Fig. 4h, i). In *t* = 9 d, interior tissues are clearly visible due to the destruction of epidermal cells by bacterial action (Fig. 4h).

In *t* = 9 d, microscopic tunnels of varying dimensions are clearly visible (Fig. 4j). According to Blanchette [36], this can be attributed to a tunnelling effect caused by *E. chuandaensis* AKC1108 in cellulosic fibril degradation. The tunnelling effect of *E. chuandaensis* AKC1108 is suspected to provide a pathway for delignification by *K. variicola* AKL1104 in inner tissues. Based on the micrograph observations, the rice straw degradation is a sequential process, where the *K. variicola* AKL1104 initiates a ligninolytic enzymatic attack on the lignin wrap and the subsequent cellulosic and hemicellulosic degradation is conducted by the *E. chuandaensis* AKC1108. The *E. chuandaensis* migrates into the inner cellulose tissue by paving access for *K. variicola* AKL1104 to internal lignin structures to degrade.

##### XRD analysis of rice straw degradation

The undegraded rice straw powder exhibited a CrI of 30.08%, which reflects an intact lignocellulosic composition dominated by crystalline cellulose encapsulated by lignin and hemicellulose wrap. On the *t* = 3 d of rice straw degradation, the CrI decreased sharply to 17.10%, which can be attributed to the lignin protective wrap degradation by *K. variicola* AKL1104 [37]. The removal of lignin via depolymerization decreases the overall recalcitrance of the rice straw, exposing hemicellulose and crystalline cellulose for subsequent bacterial degradation. A similar CrI reduction pattern was observed in rice straw degradation using *Bacillus* spp. such as *Bacillus licheniformis* due to the microscopic tunnelling effect on rice straw structure, facilitating increased accessibility to cellulosic fibres [37]. We suspect that the initiation of microscopic tunnelling caused a slight CrI decrease during degradation between *t* = 3 d and *t* = 6 d of degradation. As XRD analysis gives insights into surface topology, the *E. chuandaensis* migration into deeper cellulosic material via tunnelling might cause underrepresentation of actual CrI decrease between *t* = 3 d and *t* = 6 d and might not be ‘instrumentally-visible’ similar to the drastic CrI decrease observed in *t* = 0 d and *t* = 3 d. During the *t* = 9 d of degradation, the CrI reduced to 11.76%, indicating further cellulose hydrolysis.

The overall CrI decrease can be attributed to initial delignification followed by crystalline cellulose saccharification and crystallite cellulose fibre size reduction (95% bootstrap; CI −2.71 to −1.32; *R*² = 0.93). According to Prajapati and Kango [38], the CrI reduction from *t* = 6 d to *t* = 9 d can be attributed to cellobiohydrolases secreted from *E. chuandaensis* AKC1108 enzymatic cocktail. The CrI reduction following a rapid initial decline (from *t* = 0 d to *t* = 3 d) in bacterial rice straw degradation is consistent with previous studies by Prajapati and Kango [38] and Kumari and Singh [39]. At the molecular level, the reduction of CrI is due to hydrogen bond dissociation among polymers of the rice straw plant cell wall due to lignocellulolysis. The CrI reduction is a direct indication of the degree of polymerization of cellulose due to the consortium action.

**Fig. 5.**
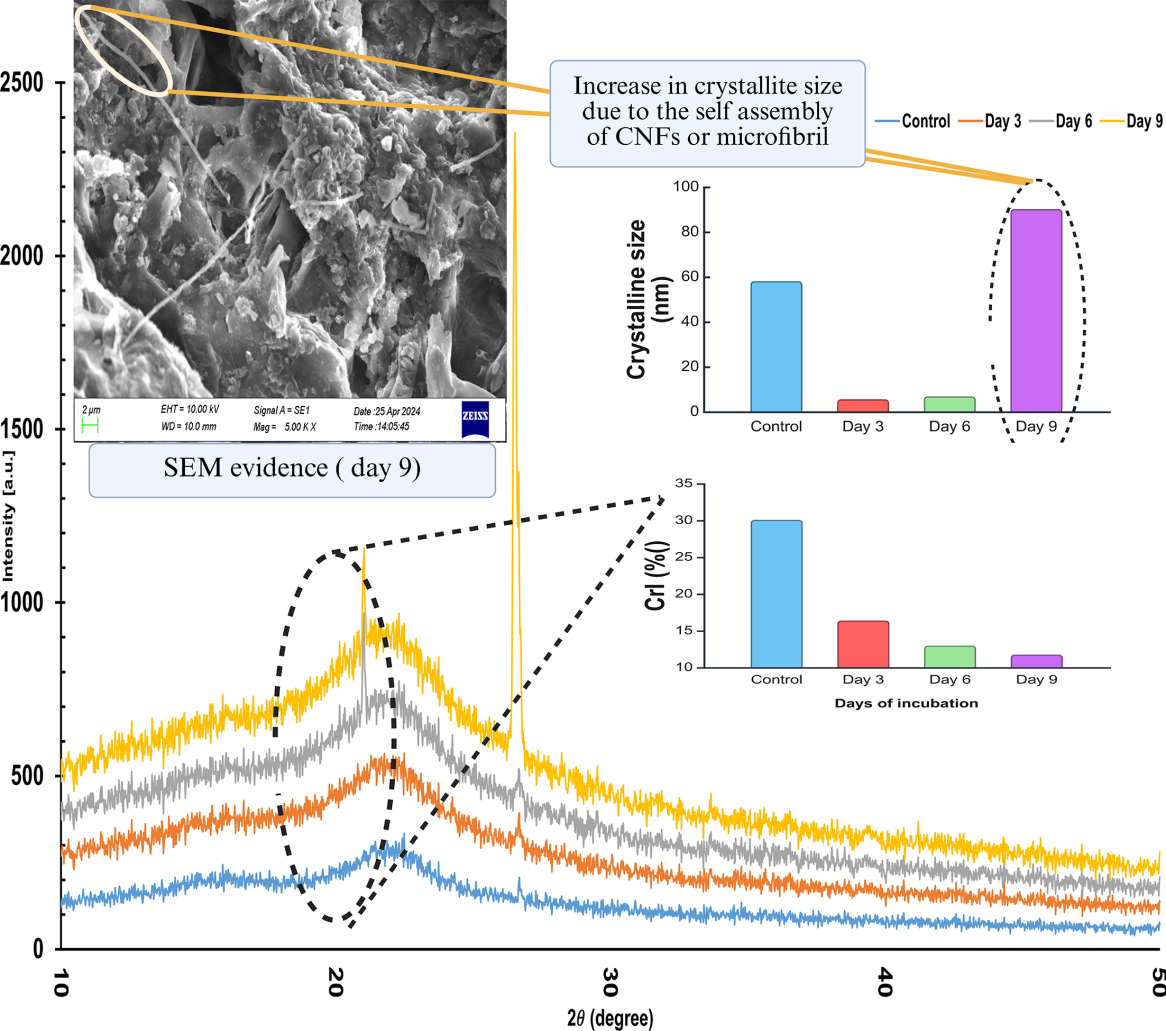
XRD graphs of rice straw biodegradation using the consortium with CrI (%) and crystallite size fluctuations. SEM evidence clearly indicates the cellulosic microfibril reaggregation, which causes a considerable CS increase.

The CS is an indicator of cellulose availability for bacterial decomposition. The CS of the cellulose fibres in undegraded rice straw is 58.11 nm (*t* = 0 d). This can be attributed to the highly ordered crystalline regions in the lignocellulosic matrix with a minimally disordered amorphous arrangement of cell walls, unaffected by the enzymatic hydrolysis. The CS of 58.11 nm indicates the presence of larger microfibrils within the plant cell wall, where smaller elementary cellulose fibrils coalesce larger microfibrils with lignin and hemicellulose. On the *t* = 3 d of rice straw biodegradation, the CS has been reduced to 5.53 nm. This sharp CS reduction indicates the removal of lignin and hemicellulose layers in the cell walls and exposes the highly ordered crystalline cellulose regions. The enzymatic attack on the cellulose chains has caused significant damage and distorted the cellulose crystals, eventually reducing the overall CS [39]. A slight increase in CS on the *t* = 6 d of rice straw biodegradation is evident with 6.81 nm CS, followed by a significant increase in the *t* = 6 d with 90.16 nm. According to Ahola *et al*. [40], the considerable increase in the CS is due to cellulose I to cellulose II recrystallization and self-assembly. The formation of long (CNF) Cellulosic Nano-fibrils-like fibrils is due to the aggregation of many ultrafine cellulosic fibres formed during cellulose hydrolysis. The presence of a higher number of carboxyl dipoles causes self-assemblage of ultrafine cellulosic fibres into larger fibres with a thin ribbon-like appearance (Fig. 4i). This cellulose fibril reaggregation is further supported by SEM observations (Fig. 4i) and FTIR analysis of *t* = 6 d and *t* = 9 d of degradation, where the absorption peaks intensity around 1,035 cm^−1^ (attributed to the C-O and O-H bonds in COOH groups substitution in cellulosic matrix during enzymatic action) is gradually increasing [40]. Prior scientific literature lacks strong evidence that the *K. variicola* and *E. chuandaensis-*mediated rice straw degradation causes a cellulose fibril reaggregation effect. Fig. 5 gives the graphical representation of CrI (%) and CS during bacterial degradation of rice straw powder. The XRD data are given in the supplementary materials. Nevertheless, the strong correlation between CrI and Cs is statistically impeded due to the self-aggregation of long CNF-like fibrils (Pearson *r* = 0.14r, *p* > 0.05; Spearman ρ = −0.20, *p* > 0.05).

Reports by Åkerholm *et al*. [41] and Nam *et al*. [42] indicate that the Scherer equation allows the calculation of the crystallite size using the (FWHM) Full Width Half-Maximum of the peak with the highest intensity of a hydrolysed cellulosic substrate. Studies suggest that the recrystallization might cause changes in crystallite size due to cellulose recrystallization during biodegradation, as recrystallization affects the interlayer distance and ultimately affects the angular position of the Bragg peak [43,44].

**Fig. 6.**
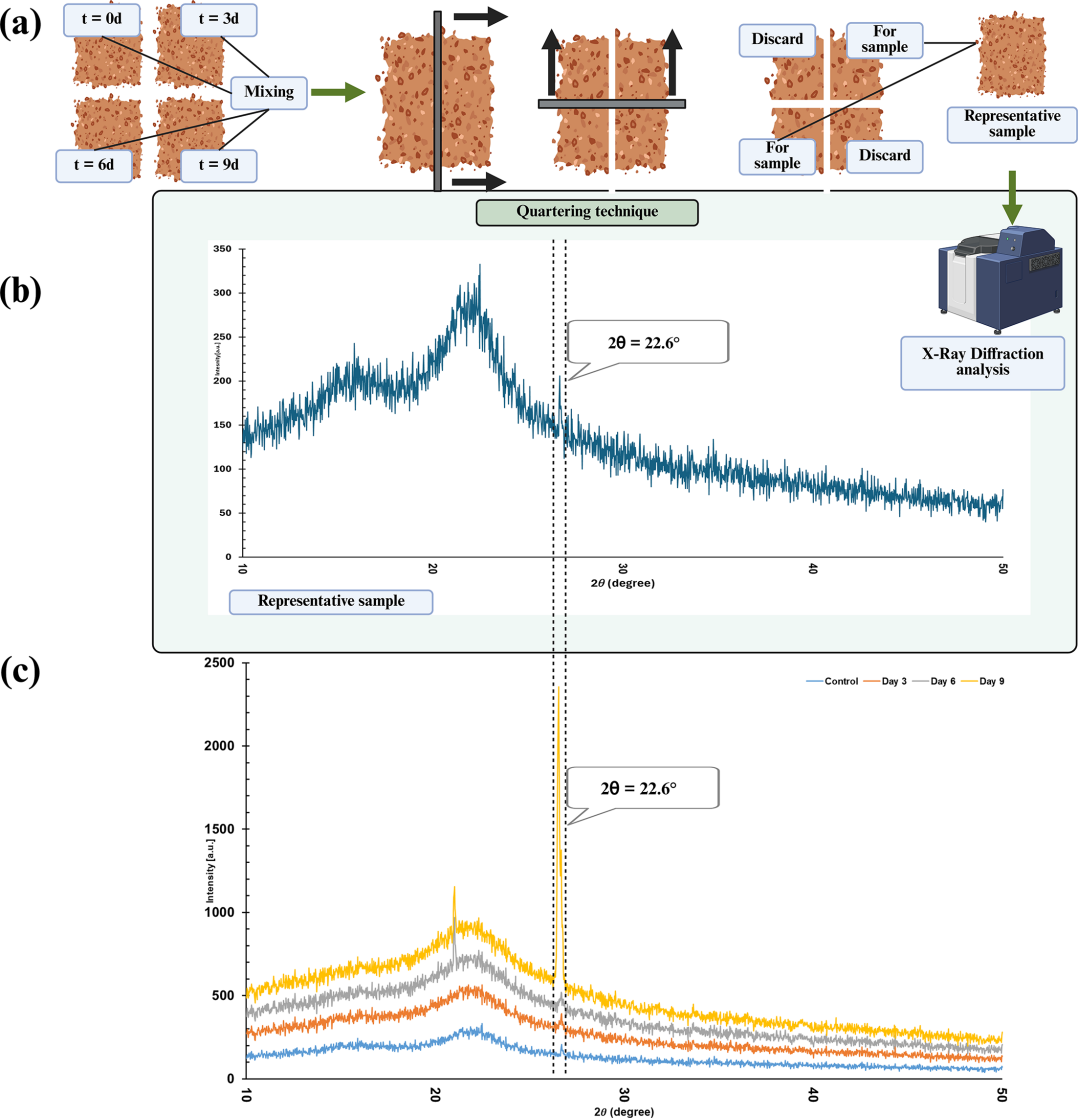
(a) The quartering protocol for acquiring the representative sample. (b) The XRD graph of the representative sample. (c) The direct comparison of the 2θ=22.6° peak represented in the prior XRD samples and the representative sample.

XRD patterns of semicrystalline substrates (rice straw) are a linear sum of crystalline and amorphous regions [45]. According to Garvey *et al*. [46], during conditions where the instrumental broadening is small (similar to the conditions used in the experiment; scan speed/duration time 2.0000 deg./min and step width 0.0200 deg.), the peak widths are inversely proportional to crystallite dimensions in the direction of the crystallographic axis. According to Ruka *et al*. [47], the peak broadening observed during progressive biodegradation of rice straw is due to the broad halo of the amorphous cellulose (Fig. 6). An increase in peak intensity similar to that observed in the study is reported by Ruka *et al*. [47]. Furthermore, reports by Vasconcelos *et al*. [48] and Ruka *et al*. [47] indicate that the 2θ=22.6° sharp peak emerging in *t* = 9 d is corresponding to cellulose Iα in rice straw. According to reports by Vasconcelos *et al*. [48], this emergence of a sharp peak at 2θ=22.6° is due to the presence of carboxymethyl moieties in nanostructure, which in return contributes to an increase in spaces between crystalline plane and eventually a considerable increase in CS (Fig. 6). Furthermore, Owen *et al*. [49] also discuss a similar peak broadening at 2θ=22.6° due to cellulose recrystallization, concluding that it leads to a larger crystallite size.

Furthermore, to confirm the observation and conclusion obtained from scientific literature, the authors used a representative sample for XRD analysis using the quartering technique [50]. The reason for the utilization of the quartering technique is to confirm the presence of the characteristic peaks observed in all the samples in the single representative sample and to rule out the possibility of any machine interference during analysis of any of the samples. The single representative sample is shown in Fig. 6. It is clear that all the characteristic XRD peaks shown in all samples are present in the representative XRD sample, including the broad and sharp peaks related to 2θ=22.6°. Therefore, it can be concluded that the broad and sharp peak at 2θ=22.6° is due to the sample characteristics at *t* = 9 d.

##### FTIR analysis of rice straw biodegradation

The rise in the S1328cm-1to1330cm-1G1269cm-1to1272cm-1 ratio from *t* = 0 d to *t* = 3 d of degradation is due to the preferential degradation of guaiacyl (G) units by *K. variicola* AKL1104 in early lignin biodegradation stages. The rise in S1328cm-1to1330cm-1G1269cm-1to1272cm-1 is attributed to fewer methoxy moieties (typically single methoxy) present in G compared to S (typically two methoxy moieties), which creates a steric hindrance to ligninolytic enzymes, making G more susceptible to initial ligninolytic enzyme degradation [51]. The decline of S1328cm-1to1330cm-1G1269cm-1to1272cm-1from *t* = 3 d to *t* = 6 d of degradation suggests the initial degradation of sterically inaccessible S-units more extensively, alongside the depletion of more sterically accessible G-units. Furthermore, the overall drop of the S1328cm-1to1330cm-1G1269cm-1to1272cm-1 ratio is an indication of lignin degradation (Table 3).

**Table 3.** S1328cm-1to1330cm-1G1269cm-1to1272cm-1ratio, IR crystallinity ratios of overall lignocellulosic degradation ($\frac{A_{{1317cm}^{-1}}}{A_{{1512cm}^{-1}}}$,$\frac{A_{{1127cm}^{-1}}}{A_{{897cm}^{-1}}}$,$\frac{A_{{2900cm}^{-1}}}{A_{{897cm}^{-1}}}$), LOI, TCI and HBI (specific for cellulosic conformational changes) during rice straw biodegradation*

	$\left(\frac{S_{1328{cm}^{-1}\;to\;1330{cm}^{-1}}}{G_{1260{cm}^{-1}\;to\;1272{cm}^{-1}}}\right)$ratio	$\left(\frac{A_{1317cm^{-1}}}{A_{1512cm^{-1}}}\right)$	$\left(\frac{A_{1127cm^{-1}}}{A_{897cm^{-1}}}\right)$	$\left(\frac{A_{2900cm^{-1}}}{A_{897cm^{-1}}}\right)$	LOI	TCI	HBI
*t* = 0 d	1.028±0.007^aC^	0.923±0.001^cD^	0.607±0.003^fA^	0.815±0.004^dA^	0.776±0.006^eA^	0.950±0.002^bA^	0.777±0.004^eA^
*t* = 3 d	1.112±0.011^aA^	0.966±0.002^cC^	0.276±0.003^gD^	0.754±0.003^dB^	0.693±0.006^eB^	0.875±0.002^bA^	0.608±0.005^fD^
*t* = 6 d	1.078±0.01^aB^	1.024±0.002^bB^	0.386±0.003^fC^	0.653±0.003^dC^	0.628±0.007^eC^	0.988±0.003^bA^	0.651±0.006^eB^
*t* = 9 d	0.960±0.006^bD^	1.195±0.002^aA^	0.529±0.003^dB^	0.639±0.002^dC^	0.503±0.007^fD^	0.945±0.001^cA^	0.577±0.005^fC^

*Ratios are presented as mean±sd. Lowercase letters indicate FTIR parameters within each incubation day, and shared lowercase letters indicate not significantly different values (Tukey’s HSD test, *α* = 0.05) within incubation time-point. Uppercase letters indicate four time-points (0 d, 3 d, 6 d and 9 d) within the same combination, and shared uppercase letters indicate no significant change over time (Tukey’s HSD test, *α* = 0.05).

The gradual increase of A1317cm-1A1512cm-1 is caused by the gradual reduction of bond intensity contributions of both syringyl peak intensities (1,317 cm^−1^) and aromatic skeletal lignin peak intensities (1,512 cm^−1^) [52] (Table 3). The overall increase of A1317cm-1A1512cm-1 from *t* = 0 d to t = 9 d of biodegradation indicates lignocellulosic biodegradation [53]. The decrease of A1127cm-1A897cm-1 from *t* = 0 d to *t* = 3 d is due to the collective reduction of peak intensities of glycosidic symmetric vibrations of C-O-C (1,127 cm^−1^) of cellulose and hemicellulose and *β*-glycosidic bond cleavage (897 cm^−1^) in cellulose due to bacterial degradation. When observed closely, the average peak intensities of 1,127 cm^−1^ (from 29.24±0.20% to 43.97±0.26%) and 897 cm^−1^ (75.64±0.32% to 83.14±0.21%) have been increased from *t* = 6 d to *t* = 9 d of biodegradation. This peak intensity increase can be attributed to the self-assemblage of cellulosic microfibrils, which is evident in CS measurements (from 6.81 nm of *t* = 6 d to 90.16 nm of *t* = 9 d of biodegradation) and SEM observations (Fig. 4). This increment in 1,127 cm^−1^ and 897 cm^−1^ peak intensities causes an overall increase of A1127cm-1A897cm-1 from *t* = 6 d to *t* = 9 d of biodegradation.

In other words, the decrease of A1127cm-1A897cm-1 from *t* = 0 d to *t* = 3 d of biodegradation and subsequent increase from *t* = 6d to *t* = 9 d of biodegradation is consistent with CrI and CS observations and provides further evidence of cellulosic microfibrils self-assemblage during *t* = 6 d to *t* = 9 d of biodegradation. The A2900cm-1A897cm-1 ratio is indicating a similar trend of intensity reduction in the 2,900 cm^−1^ wavelength region, which corresponds to C-H stretching of crystalline cellulose from day *t* = 0 to *t* = 3 d of biodegradation and an increase from *t* = 6 to *t* = 9 d of biodegradation due to the self-assembly of CNF-like fibrils observed in SEM (Fig. 3i) and evident in XRD observations from *t* = 6 d to *t* = 9 d of biodegradation. However, the A2900cm-1A897cm-1 is showing a gradual reduction indicating overall cellulosic degradation (Table 3). This indicates the cellulose degradation rate is higher than the cellulosic microfibrils’ self-assembly rate during day *t* = 6 d and *t* = 9 d of biodegradation. However, to investigate further into the changes occurring in cellulosic compounds in the cell wall, IR crystallinity ratios specific to cellulosic compounds were used.

The LOI is subjected to a gradual decrease from *t* = 0 d to *t* = 9 d of biodegradation. LOI is an indicator of the overall molecular order of cellulose (Table 3). The gradual reduction of LOI can be attributed to the progressive disruption of crystalline cellulose and an increase in amorphous cellulose content. Furthermore, the gradual LOI reduction indicates the presence of fewer hydrogen bonds in cellulose fibrils and weakly ordered cellulose conformation [54].

The TCI has shown an overall reduction at *t* = 9 d compared to *t* = 0 d. The overall reduction of TCI can be attributed to the bioconversion of cellulose I (native cellulose) to cellulose II by bacterial enzymatic action [55] (Table 3). This is consistent with FTIR and CS calculations. The overall decrease in TCI causes surface accessibility enhancements and theoretically enhances efficient cellulose hydrolysis. The sudden spike in TCI observed in *t* = 6 d of biodegradation is caused by exposure of novel crystallite cellulose regions after the lignin wrap is removed via oxidation and hydrolysis by *K. variicola* AKL1104 and during rice straw for biodegradation.

**Fig. 7.**
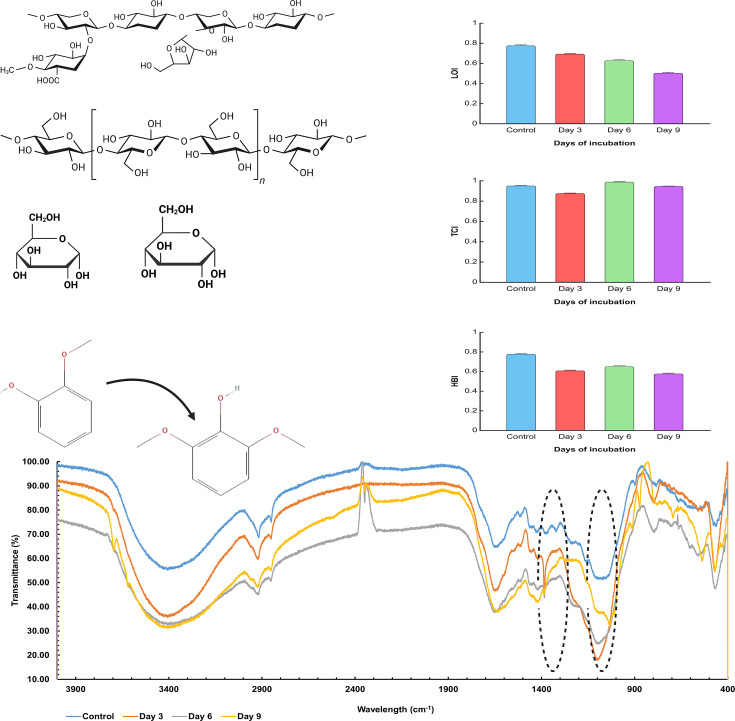
FTIR analysis of rice straw degradation with LOI (**a**), TCI (**b**) and HBI (**c**) fluctuations. This increment in HBI and LCI in day t=6 d is reduced in t=9 d, indicating the further degradation of newly exposed crystallite cellulose in deeper tissues of rice straw. An overview of S1328cm-1to1330cm-1G1269cm-1to1272cm-1 ratio, IR crystallinity ratios of overall lignocellulosic degradation ($\frac{A_{{1317cm}^{-1}}}{A_{{1512cm}^{-1}}}$,$\frac{A_{{1127cm}^{-1}}}{A_{{897cm}^{-1}}}$,$\frac{A_{{2900cm}^{-1}}}{A_{{897cm}^{-1}}}$), LOI, TCI and HBI (specific for cellulosic conformational changes) during rice straw biodegradation.

HBI reduction indicates the inter- and intramolecular hydrogen bond cleavage in crystalline cellulose and crystalline cellulose transformation into amorphous cellulose due to bacterial degradation (Table 3). According to Xiao *et al*. [56], HBI reduction is a direct indication of an increasing degree of intermolecular irregularity in cell walls due to bacterial degradation of rice straw. Similar to TCI, a considerable HBI increase at *t* = 6 d of biodegradation is evident, which can be attributed to the exposure of novel crystallite cellulose regions in deeper rice straw tissues later than the surface ligninolysis. Fig. 7 gives the FTIR spectra, LOI, TCI and HBI during biodegradation. FTIR data are given in the supplementary materials.

##### XPS analysis of rice straw biodegradation

The XPS spectra indicate that the primary elements in the undegraded rice straw powder surface comprise C, O and N, which is characteristic of lignocellulosic materials. The increase in O% and the OC ratio indicates the oxidative and hydrolysis due to *K. variicola* AKL1104 and *E. chuandaensis* AKC1108. This O% and OC ratio increase also signals the rapid removal of terpenes, lignin and fatty acid compounds from rice straw surface.

The rapid rise of O% from *t* = 0 d to *t* = 3 d can be attributed to extensive lignin oxidation due to *K. variicola* AKL1104 action. This observation is consistent with previous studies by Huang *et al*. [57], Nzokou and Kamdem [58] and Popescu *et al*. [59] on lignocellulose degradation. The C% is at 75% in undegraded rice straw powder, and a higher C% is characteristic of intact lignocellulosic surfaces without any oxidation. The rapid reduction of C% (~20% decrease) from *t* = 0 d to *t* = 3 d of biodegradation can be attributed to extensive lignin oxidation in cell walls by *K. variicola* AKL1104. However, in *t* = 6 d, the C% increases, signalling the exposure of crystalline cellulosic regions after lignin wrap removal. Furthermore, the overall reduction of C% and O% from *t* = 0 d to *t* = 9 d indicates the oxidative and hydrolytic removal of polysaccharides from cell walls. The CN is an indicator ratio of lignocellulosic material degradation. The overall reduction of the CN ratio of undegraded rice straw from *t* = 0 d to *t* = 9 d of biodegradation indicates the rapid lignocellulolysis. The fluctuations (small increments in CN ratio) during day *t* = 6 d and *t* = 9 d of biodegradation are due to interference on the N_1s_ signal caused by microbial colonization and migration of bacterial cells within the matrix [60,61]. The N% in undegraded rice straw can be attributed to N_1s_ peak of nitrogenous compounds corresponding to C–(NH)–C, amide compounds (O=C–N–C/H–N–H) and C=N–C) [62]. Table 4 presents the C%, O%, N%, $\frac{O}{C}$ ratio and *t* = 9 ratio fluctuations during rice straw degradation.

**Table 4.** C%, O% and N% contributions and OCand CN ratio fluctuations during consortium-mediated rice straw biodegradation*

	C%	O%	N%	$\frac{O}{C}$	$\frac{C}{N}$
*t* = 0 d	75.0	22.05	2.94	0.60	20.50
*t* = 3 d	55.02	39.71	5.26	0.40	18.45
*t* = 6 d	57.89	37.36	4.73	0.64	12.22
*t* = 9 d	51.89	44.32	3.78	0.85	13.71

*The spectra of Si_2p_ were discarded as the lignocellulosic biodegradation focused on organic matter degradation.

##### N_1s_ spectra give insights into the mechanisms of bacterial consortia attachment to the substrate

**Fig. 8.**
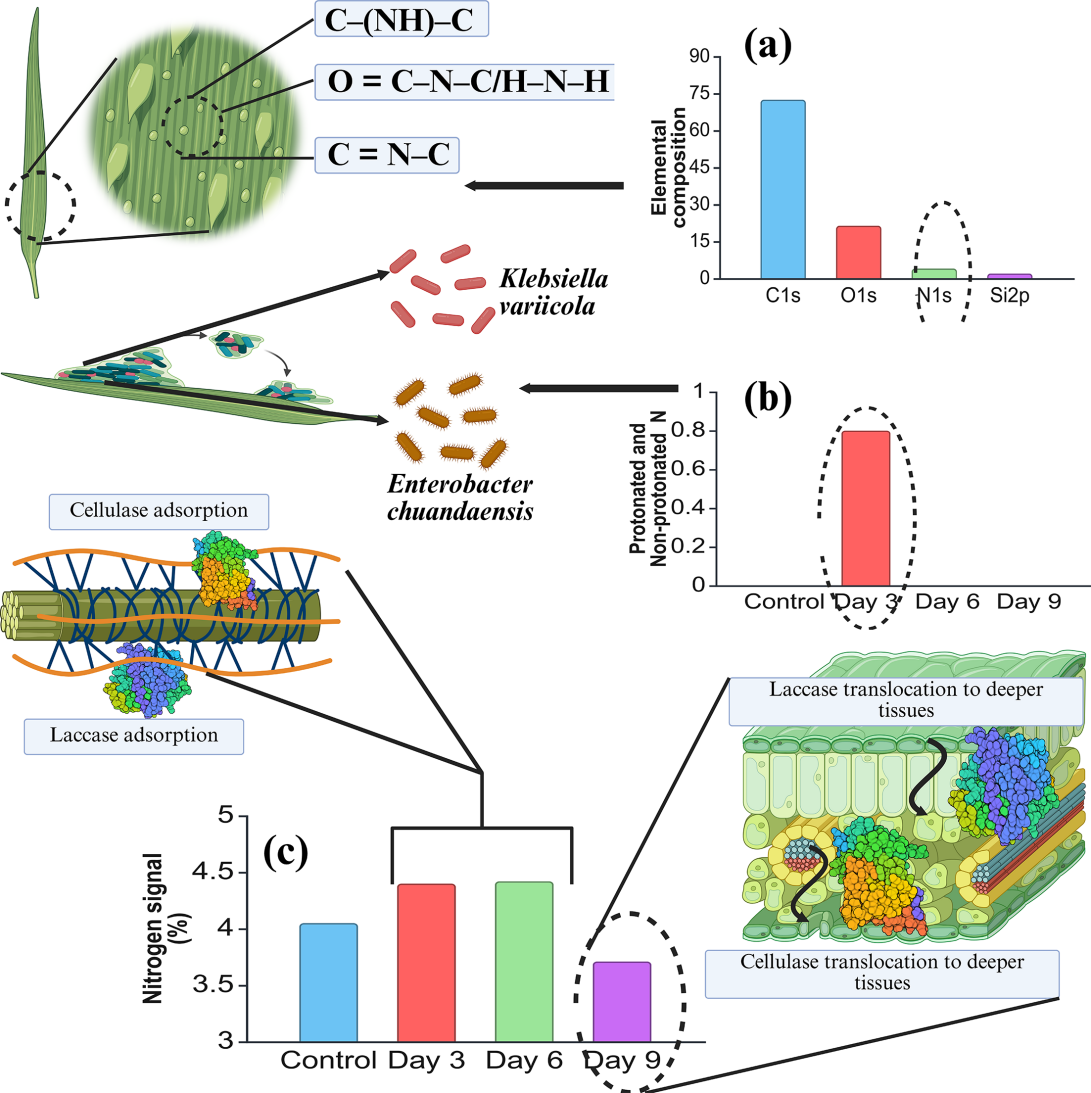
Mechanism of colonization of the consortium elucidated using N_1s_ XPS data. Protein structures retrieved from the Protein Data Bank.

The considerable N% increase during the *t* = 3 d confirms biomass accumulation due to bacterial colonization and attachment on the rice straw powder surface. The peaks emerging from N_1s_ at 404.91 eV are due to the characteristic N _non-protonated_ (non-protonated N) and N_protonated_ (protonated N) from bacterial cell surfaces of *K. variicola* AKL1104 and *E. chuandaensis* AKC1108 [60]. The trend of elevated N% throughout the degradation compared to the control can be attributed to bacterial colonization and attachment to the straw surface. A new insight into the sudden increase in N% during initiation (from *t* = 0 d to *t* = 3 d) of biodegradation and throughout the degradation is given by Corrales-Ureña *et al*. [63]. The considerable N% increase during initiation of biodegradation and throughout the biodegradation period might be due to stable adsorption of laccase from *K. variicola* AKL1104 and cellulases from *E. chuandaensis* AKC1108 to the lignin surface, and the gradual drop of N% during the biodegradation after *t* = 9 d might be due to laccases and cellulase translocation along the lignin surface and cellulose fibrils. A sudden increase in N% during initiation (from *t* = 0 d to *t* = 3 d) might signal *E. chuandaensis* AKC1108 migration into the deeper tissues targeting amorphous cellulosic regions due to its tunnelling effect on cellulose (Fig. 8).

##### C_1s_ spectra: surface oxidation reveals lignin-to-cellulose degradation dynamics

The C_1s_ signal of the XPS spectrum consists of four peaks after deconvolution, corresponding to the C_1_((C–(C, H)), C_2_ (C–O), C_3_ (C=O(O–C–O)) and C_4_ (COOH) [62,63]. The C_1_ peak corresponds to the non-functionalized C situated in lignin aliphatic side chains and aromatic rings. The overall C_1_ peak intensity reduction from *t* = 0 d to *t* = 9 d is due to lignin depolymerization due to *K. variicola* AKC1104 action on rice straw surface. A considerable C_1_ peak intensity reduction from *t* = 0 d to *t* = 3 d is observable (Table 5). This is a definitive indication of initial lignin wrap depolymerization by *K. variicola* AKL1104 as a part of a sequential degradation strategy evident in SEM, FTIR and XRD analysis results. The sudden C_1_ peak intensity increase from *t* = 3 d to *t* = 6 d might be due to the contribution of CH_2_ wagging of crystalline cellulose exposed after lignin wrap removal. The eventual decrease from *t* = 6 d to *t* = 9 d is due to the residual lignin extractives (remnants of the initial lignin depolymerization) degradation by *K. variicola* AKL1104. C_2_ (C–O) peak is primarily derived from cellulose and lignin (Table 5). The relative contribution of the C_2_ (C–O) peak intensity in the C_1s_ spectra is subjected to a gradual decrease throughout the biodegradation process. The overall suggestive reduction of C_2_ (C–O) peak intensity can be attributed to the aliphatic hydroxyl group hydrolysis by *K. variicola* AKL1104 and *β*-1,4 glycosidic bond cleavage of cellulose by *E. chuandaensis* AKC1108 in a sequential mechanism [64]. The C_2_ (C–O) indicates the stabilization of the relative peak contribution % of C_2_ (C–O) observed during *t* = 3 d and *t* = 6 d is the phase where the surface biodegradation primarily switches from lignin and hemicellulose degradation to cellulose degradation [65] (Table 5). The drastic reduction of the relative peak contribution % of C_2_ (C–O) from *t* = 6 d to *t* = 9 d is due to the rapid cleavage of *β*-1,4 glycosidic bonds in crystalline cellulose and the transformation of crystalline regions into amorphous cellulosic regions by *E. chuandaensis* AKC1108 via progressive elimination of glucose moieties from reducing ends of glucose chains (Table 5).

**Table 5.** The proportion of relative contribution % of bonds C_1_((C–(C, H), C_2_(C–O), C_3_(C=O(O–C–O)) and C_4_(COOH) and ratio of C–O / [C=O(O–C–O) + COOH]; CoxygenatedCunoxygenated ratio and acid/base microenvironment fluctuations during rice biodegradation

	Proportion relative contribution of bonds (%)				Ratio of C–O / [C=O(O–C– + COOH]	$\frac{C_{oxygenated}}{C_{unoxygenated}}$ratio	$\frac{\left(C_{2}+C_{4}\right)}{\left(C_{1}+C_{3}\right)}$ ratio	SLC%*ą
	C_1_ (C–(C,H)	C_2_(C–O)^*^	C_3_ (C=O(O–C–O))	C_4_ (COOH)				
*t* = 0 d	29.41	23.52	7.35	14.70	1.06	1.55	1.04	5.01
*t* =3 d	14.35	18.18	9.09	1.91	1.65	2.03	0.85	2.66
*t* = 6 d	19.47	19.47	8.42	10.52	1.02	1.97	1.07	2.43
*t* = 9 d	16.21	10.27	16.21	9.18	0.40	2.20	0.6	2.20

*C_2_ (C–O) and SLC% decrease with (*Mann Kendall S* = –6; p<0.05) with a monotonic trend; ą, recalculated values of SLC% from Corrected $\frac{O}{C}$-based estimation reveals progressive SLC loss section.

The increase in the relative peak contribution % of C_3_ (C=O(O–C–O)) from *t* = 0 d to *t* = 3 d is caused by oxidative lignin side chains degradation on the rice straw surface. This increase is caused by *β*-O-4, *β*-5 bond cleavage in lignins and exposure of C–O and C=O bonds [65] (Table 5). The stabilization of the relative peak contribution % of C_3_ (C=O(O–C–O)) during *t* = 3 d and *t* = 6 d of the primary biodegradation reaction represents the switch from oxidative cleavage of lignin and hemicellulose to cellulose oxidtion (Table 5). The drastic increase in the relative peak contribution % of C_3_ (C=O(O–C–O)) from *t* = 6 d to *t* = 9 d indicates extensive cellulosic oxidation, forming carbonyl end groups and/or acetal moieties during crystalline cellulose to amorphous cellulose biotransformation (Table 5). According to Csiszár and Fekete [66], the increase in the relative contribution % of C_3_ (C=O(O–C–O)) is a direct indicator of gradual lignin removal from the rice straw surface. Furthermore, the overall increase in relative contribution % of C_3_ (C=O(O–C–O)) from *t* = 0 d to *t* = 9 d can be attributed to the gradual increase in cellulosic and hemicellulosic extractives in the cell surface. Nevertheless, since hemicelluloses and lignin extractives also contribute to the C_3_ (C=O(O–C–O)), it is challenging to attribute the changes of C_3_ (C=O(O–C–O)) entirely to oxidative cleavage of cellulose [67,68].

The strong presence of C_4_ (COOH) in the undegraded rice straw powder in *t* = 0 d suggests the presence of lignin and hemicellulosic wrap encasing cellulose in cell walls. The considerable relative peak contribution % decrease of C_4_ (COOH) from *t* = 0 d to of *t* = 3 d indicates complete lignin wrap removal, encasing cell walls by *K. variicola* AKL1104 action via O–C bond cleavage in lignin (Table 5). Furthermore, a collective contribution from hemicellulosic removal and lignin metabolites oxidation may also influence this considerable decrease of relative peak contribution % of C_4_ (COOH). Apart from lignin degradation, the accumulation of the hydrocarbons from lignin and hemicellulose degradation on the degradation site might impede the XPS signal from C_4_ COOH and can actively contribute to the considerable decrease of relative peak contribution % of C_4_ (COOH) observed at *t* = 3 d [69]. After the hydrocarbons removal from rice straw surface via progressive degradation of accumulated hydrocarbons by the bacterial consortia, the relative peak contribution % of C_4_ (COOH) started reappearing in *t* = 6 d and decreasing from t = 6 d to *t* = 9 d (Table 5). These observations are consistent with FTIR analysis of rice straw degradation.

The ratio of C–O / [C=O(O–C–O) + COOH] has increased from *t* = 0 d to *t* = 3 d which is caused by extensive lignin and hemicellulosic encapsulation removal around cellulose, and the value of C–O / [C=O(O–C–O) + COOH] ratio gradually reduces from *t* = 3 d to *t* = 9 d of biodegradation indicating gradual decomposition of cellulose and residual hydrocarbons. Table 5 summarizes the proportion of relative contribution % of bonds C_1_((C–(C, H), C_2_(C–O), C_3_(C=O(O–C–O)), C_4_(COOH) and ratio of C–O / [C=O(O–C–O) + COOH] during biodegradation. Due to the presence of the C_4_ (COOH) signal in the XPS spectrum, the equation for calculating the CoxygenatedCunoxygenated ratio ($C_{0xygenated}$ = oxidized C_1s_ carbon, $C_{unoxygenated}$ = unoxidized C_1s_ carbon) was modified as follows to incorporate the influence of C_4_ (COOH) signal [70,71].


Coxygenated/Cunoxygenated=C2+C3+C4C1(9)

The CoxygenatedCunoxygenated ratio is an indicator of the extent of oxidation that occurred on the rice straw surface. The overall increase in the CoxygenatedCunoxygenated ratio is due to the oxidation and hydrolysis of cell walls due to consortium-mediated rice straw biodegradation. The increase from *t* = 0 d to *t* = 3 d can be attributed to the extensive oxidation and hydrolysis of lignin and hemicellulose by *K. variicola* AKL1104, followed by gradual stabilization of the CoxygenatedCunoxygenated ratio from *t* = 3 d to *t* = 6 d indicating that switching the biodegradation strategy from lignin degradation to cellulose degradation with *E. chuandaensis* AKC1108 and the further increase of CoxygenatedCunoxygenated ratio from *t* = 6 d to *t* = 9 d is caused by the cellulose oxidization of *E. chuandaensis* AKC1108. Table 5 summarizes the CoxygenatedCunoxygenatedratio during rice straw biodegradation.

The acid/base balance in the undegraded rice straw is 1.04, indicating neutrality of acidic and basic moieties on the rice straw surface. The acid/base balance shifts from neutrality to an alkaline microenvironment (0.85) due to the extensive -O-4, *β*-5 bond cleavage in lignins and exposure of C–O and C=O bonds during *t* = 3 d. The acid/base balance reaches neutrality (1.07) in *t* = 6 d due to the exposure of the crystalline and amorphous cellulosic regions after the removal of lignin and hemicellulosic wrap around cell walls. The acid/base balance further shifts to alkaline environments due to the accumulation of electron acceptors (bases) on the rice straw surface caused by the collective action of oxidation of cellulosic surfaces forming carbonyl end groups and/or acetal moieties during biotransformation of crystalline cellulose to amorphous cellulose. Table 5 gives a summary of acid/base microenvironment fluctuations during rice straw degradation.

##### O_1s_ deconvolution maps lignin-to-cellulose transition

The O_1s_ exhibited two sub-spectra between 545 and 525 eV. The O_1_ spectra near the maximum binding energy of 530.51 eV are attributed to O atoms in O–C=O of lignin on the rice straw surface. The O_2_ spectra near maximum binding energy of 529 eV correspond to the O atoms in C– O– bonds of cellulose and hemicellulose. The relative peak contribution % of O_1_ (86.66%) spectra is considerably higher than the relative peak contribution % of O_2_ spectra (13.33%), indicating a complete and intact lignin wrap around the cellulose of cell walls. The relative peak contribution of % O_1_ is decreasing from *t* = 0 d to *t* = 3 d during biodegradation due to the complete lignin wrap removal due to *K. variicola* AKL1104 action (Table 6). However, the residual lignin short chains accumulation and carbohydrate content hydrolysis on the straw surface is causing a collective increase of relative peak contribution % of O_1_ from *t* = 3 d to *t* = 9 d (Table 6). This observation is consistent with acid/base fluctuations within the rice straw microenvironments, which shift to alkalinity.

**Table 6.** The proportion of relative contribution % of bonds of O_1_(O–C=O), O_2_(C–O) and O_3_ (C=O) microenvironment fluctuations during rice biodegradation

	Proportion relative contribution of bonds (%)		
	O_1_ (O–C=O)	O_2_ (C–O)	O_3_ (C=O)
*t* = 0 d	19.11	2.94	0.00*
*t* = 3 d	15.78	9.09	14.83
*t* = 6 d	16.31	12.10	8.94
*t* = 9 d	23.24	8.64	12.43

*No signal detected.

The relative peak contribution % of the O_2_ signal corresponding to the (C–O) cellulose in cell walls is minimum (*t* = 0 d). This is due to the masking of cellulose by the lignin wrap in cell walls. The considerable increase in relative peak contribution % of the O_2_ peak from *t* = 0 d to of t=3 d (9.09%) is due to the exposure of cellulose following the complete removal of lignin wrap by *K. variicola* AKL1104. The gradual increase from *t* = 6 d to *t* = 9 d is further attributed to the removal of cellulosic material due to the cellulolytic action of *E. chuandaensis* AKC1108. Nevertheless, the sudden decrease in the relative contribution % of the O_2_ signal might be due to the rapid cleavage of *β*-1,4 glycosidic bonds in crystalline cellulose and the transformation of crystalline regions into amorphous cellulosic regions by *E. chuandaensis* AKC1108 via progressive elimination of glucose moieties from the reducing end of glucose chains. This is consistent in bond intensity fluctuations of C_2_ (C–O) sub-spectra in the C_1s_ spectrum.

During *t* = 3 d, an additional O_3_ (C=O) sub-spectrum with considerable relative peak contribution % appeared in the O_1s_ spectrum (Table 6). This peak is due to the phenolic O formed due to *β*-O-4 link cleavage by *K. variicola* AKL1104, exposing phenolic hydroxyl moieties on the rice straw surface. The reduction of the O_3_(C=O) peak intensity % in *t* = 6 d is due to the oxidation of terminal hydroxymethyl and phenolic end-groups to carbonyl moieties originating from already cleaved lignin main chains (Table 6). This also can be a cause for the considerable increase in relative contribution % of C_4_ (COOH) from *t* = 3 d to *t* = 6 d and the observed reduction of relative contribution % of C_3_ (C=O(O–C–O)) from *t* = 3 d to *t* = 6 d due to the oxidative removal of ketonic or aldehyde intermediates during the oxidation of terminal hydroxymethyl and phenolic end-groups to carbonyl moieties [72]. The rise of the O_3_(C=O) peak intensity from *t* = 6 d to *t* = 9 d is due to the further biodegradation of lignin side chains into shorter chains by *K. variicola* AKL1104 action (Table 6).

##### Corrected OC-based estimation reveals progressive SLC loss

The approach used by Liang *et al*. [25] for SLC estimation gives erroneous values for the samples. Furthermore, the approach used by Laine *et al*. [73] and Ström and Carlsson [74] also delivers erroneous results for SLC. In Equation (6), theNo(sample)Nc(sample) is the OC ratio of the sample, and the No(cellulose)Nc(cellulose) is the OC ratio of cellulose, while No(lignin)Nc(lignin)is the OC ratio of lignin. The resultant value, when computed using the measured values of the sample, is erroneous. This issue with Equation (6) and the approach by Liang *et al*. [25] was first highlighted by Li and Reeve [75], and a linear relationship between surface lignin coverage and No(sample)Nc(sample) cannot be established. The equation proposed by Liang *et al*. [25] (earlier by Laine *et al*. [73] and Ström and Carlsson [74]) provides a generalized overview of where an empirical formula for polysaccharide and lignin counterparts of the rice straw fibre is utilized to estimate the SLC. For carbohydrate with molecular formula of $C_{m}O_{n}$ and molecular formula of lignin with $C_{x}O_{y}$, the mole fraction ($S_{lignin})$) and weight fraction ($W_{lignin})$) of lignin are as follows:


Slignin=n-mNoNcn-y+(x-m)NoNc(10)

Wlignin=MligninMcarbohydrateSlignin1-Mcarbohydrate-MligninMcarbohydrateSlignin(11)

$M_{lignin}$ and $M_{carbohydrates}$ are the molar masses of lignin and carbohydrates on the rice straw surface. The $M_{lignin}$=12m+6 n and $M_{carbohydrate}=12x+6y$ by Li and Reeve [75] are erroneous because the H atoms in lignin and other carbohydrates do not contribute to the XPS spectra but carry a mass. Therefore, the segment molar masses should be incorporated into Equation (11) to calculate the $W_{lignin}.$ Major erroneous calculations that emerge in SLC from Equation (6) in low lignin compounds, such as rice straw and during lignin degradation.

The NoNc value of 0.83 of pure cellulose is rarely observed in experimental conditions due to the contamination interference from the presence of carbon-rich moieties, such as lignin oligomers and during lignin degradation. The presence of lignin compounds should be considered during SLC calculation. Nevertheless, the experimental conditions for absolute XPS readings for atomic ratios are challenging to achieve and implicit or explicit calibration approaches are used against a reference material with pure cellulose, such as filter paper. For example, Equation (6) assumes a linear algebraic relationship between SLC and NoNc for pure cellulose. Therefore, we propose a correction method that comprises a few assumptions. The samples are assumed to contain only lignin, cellulose and hemicellulose on the surface, while the lignocellulosic extractives are removed due to biodegradation by bacteria.

1. The lignocellulosic composition of the analysed material in close proximity to the surface is uniform.
2. The polysaccharides in the surface layer are represented by empirical chemical composition of $C_{6}O_{5}$ and 162.1 as molar mass.
3. The empirical formula for lignin is $C_{9.92}O_{3.32}$ with a molar mass of 183.5.
4. Lignin is considered as the excess C signal originating from the XPS spectra of rice straw surface, which is comprised only of cellulose and hemicellulose.

To obtain the observed OC ratio (NoNc)) of the surface composition, it is assumed that during the XPS sampling process, there were S number of anhydroglucose or sugar units and L number of lignin phenylpropane segments per unit volume of rice straw powder sampled at the surface. From the empirical relationship for S and L, the number of O and C atoms in the rice straw volume was as follows:


$$N_{o}=5S+3.32L(12)$$

And


$$N_{C}=6S+9.92L(13)$$

The SLC was obtained from LL+S. From the empirical relationship of Sand L, the SLC is given by


SLC=LL+S=5-6NONC1.68+3.92NONC(14)

The $W_{lignin}$ is given by


$$W_{lignin}=\frac{1.132S_{L}}{1+0.132S_{L}}(15)$$

We use the following approach to rectify the measured OC ratios for the *excess* C signal arising due to lignin spreading on the rice straw and lignin degradation in XPS spectra. For pure cellulose, the NONC is 5/6 or 0.833. The reported NONC of derived cellulose from rice straw is 0.74 [76]. This lower value can be attributed to C1 spectra contribution from C atoms covalently bonded to O. The primary assumption is that the excess C signal in the rice straw-derived samples, which is in the same proportion to the total NONC as is the C contribution signal: C signal of measured rice straw samples. The excess C signal due to the presence of lignin is expressed as follows:


NCexcessNC+NOricestrawcellulose=NCexcessNC+NOricestrawsample(16)

The contribution from lignin to the C1 signal makes an equivalent contribution to the total XPS spectrum for both rice straw cellulose and the rice straw sample containing lignin. This is a reasonable assumption for lignocellulosic materials with similar cellulosic structures. The NCexcessNCNO for rice straw cellulose is expressed as follows.


$${\left(\frac{N_{O}}{N_{C}}\right)}_{cellulose\;theoretical\;}=C_{theoretical}=0.833$$

NONCricestrawcellulose=Cricestrawcellulose

NONCricestrawmeasured=Cricestrawsample(measured)

NONCricestrawsample(corrected)=Cricestrawsample(corrected)

This adheres to the usual convention of values reporting of XPS spectra from individual elements. The OC ratio for rice straw cellulose comprises added contributions from lignin contamination.


Cricestrawcellulose=NONC+NCexcess(17)

Hence,


(18)NcexcessNo=1Crice straw cellulose(Ctheoretical−Crice straw celluloseCtheoretical+1)=1NoNcCrice straw cellulose(0.833−(NoNc)Crice straw cellulose1.833)

The NCexcessNCNO value for rice straw cellulose assumes that Equation (18) will approximate the $\frac{N_{c}^{\text{excess}}}{\left(\frac{N_{c}}{N_{o}}\right)}$ value for rice straw samples at the same time as pure cellulose standard. Explaining further, the rice straw samples and rice straw-derived cellulose both contain lignin contamination. To rectify the excess contribution from C from lignin, the corrected value of $N_{C}^{excess}$ and the corrected OC ratio for rice straw samples are given by


(19)NoNcrice straw sample, corrected=Crice straw sample (corrected)=NO rice straw sample, measuredNC rice straw sample (measured)−NCexcess

Therefore,


$$\frac{N_{C}^{excess}}{N_{C}+N_{O}}=1-\frac{\left(\frac{1}{C_{ricestrawsample\left(corrected\right)}}\right)+1}{\left(\frac{1}{C_{ricestrawsample\left(measured\right)}}\right)+1}(20)$$

Hence,


$$\frac{1}{C_{ricestrawsample(corrected)}}=\left\{\left\{\left(1-\frac{N_{C}^{excess}}{\left(N_{C}+N_{O}\right)}\right)\right\}\left(\frac{1}{C_{ricestrawsample(measured)}}+1\right)\right\}-1(21)$$

By combining Equations (19) and (20),


(22) 1NONCricestrawsample,corrected=1+NONCricestrawcellulose,measured1.8331NONCricestrawsample,measured+1-1

From this equation, we calculated the corrected OC ratio NONCricestrawsample,corrected. When observed closely, the NONCricestrawsample,corrected values are higher than the NONCricestrawsample,measured values for rice straw samples. Therefore, it can be concluded that the $N_{C}^{excess}$ is caused by NONClignin of lignin on the rice straw surface. It can be expressed as simple algebraic form as follows:

NONCricestrawsample,corrected=NONCricestrawsample,measure+NONClignin (23).

Therefore,


(24) NONClignin=NONCricestrawsample,corrected-NONCricestrawsample,measured

The SLC % is interpreted as follows:


SLC%=NONCligninNONCricestrawsample,corrected×100(25)

The recalculated NONClignin is as follows during the biodegradation in Table 7. Further validation of the new equation is conducted experimentally in the next phase of the research.

**Table 7.** The NONClignin contribution to rice straw samples

	${\left(\frac{N_{O}}{N_{C}}\right)}_{rice\;straw\;sample,\;corrected}$	${\left(\frac{N_{O}}{N_{C}}\right)}_{rice\;straw\;sample,\;measured}$	${\left(\frac{N_{O}}{N_{C}}\right)}_{lignin}$
*t* = 0 d	0.66	0.60	0.05
*t* = 3 d	0.44	0.40	0.03
*t* = 6 d	0.70	0.64	0.05
*t* =9 d	0.94	0.85	0.08

**Fig. 9.**
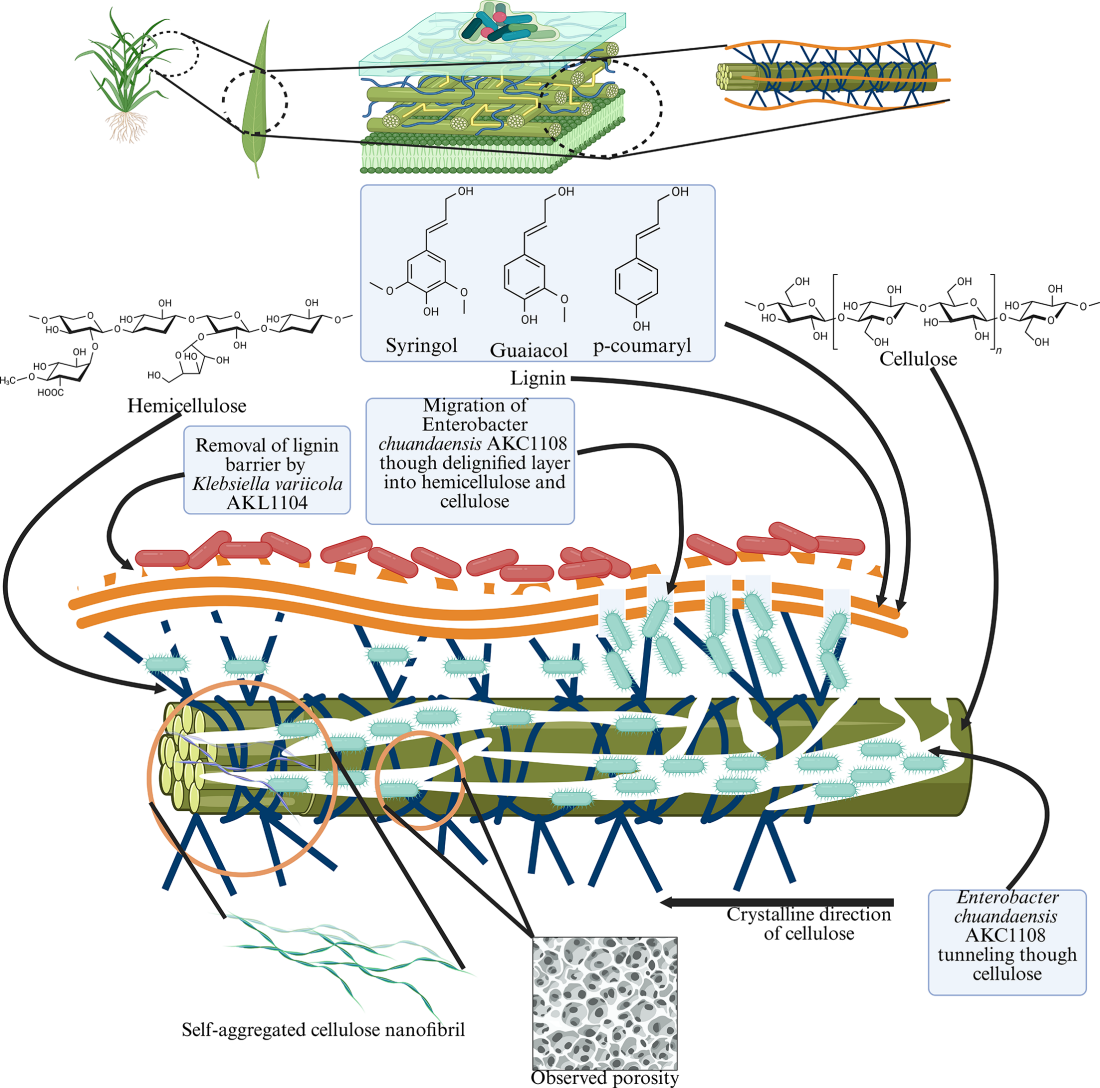
Graphic illustration of the suggested combinatorial mechanism of rice straw degradation.

The SLC % during biodegradation is computed according to the modified equation, as given in Table 5. The rapid decrease of SLC% from *t* = 0 d to *t* = 3 d (~46.90% decrease) is due to the complete lignin removal due to the action of *K. variicola* AKL1104 in the consortium (CI –0.78 to –0.08; S = –6, *p* < 0.05). This sharp decline from *t* = 0 d to *t* = 9 d is evident in C1s XPS spectra and FTIR results. The gradual decline of SLC % from *t* = 3 d to *t* = 9 d confirms the gradual removal of surface lignin during bacterial biodegradation. The XPS spectrum data of C_1s_, O_1s_ and N_1s_ elements during bacterial biodegradation of rice straw are in the supplementary materials. The graphical representation of the proposed mechanism of rice straw degradation is shown in Fig. 9.

## Conclusion

The combinatorial mechanism of rice straw degradation by the ligninolytic *K. variicola* and cellulolytic *E. chuandaensis* is performed sequentially. First, the lignin barrier is removed by the ligninolytic *K. variicola* in the early days of biodegradation, and the migration of the cellulolytic *E. chuandaensis* into the inner hemicellulose and cellulose material conducts cellulose degradation. The *E. chuandaensis* migrates deeper into the cellulose material in the cell wall, creating porous structures on the rice straw surface. The driving force behind the successful synergy between both isolates is the co-evolution within the rice straw tissues. This confirms the notion that the endophytic isolates are playing a key role in lignocellulose biodegradation. The division of labour among *K. variicola* and *E. chuandaensis* eases the metabolic burden on the bacterial community during the coordinated attack on lignin, followed by secondary attack on cellulose. Future steps of the research include further validations for the corrections imposed on the SLC measurement equation through experimental approaches. The metabolites of biodegradation and further elucidation of the suspected mechanism of biodegradation should be examined. Prior to the consortium deployment to the environment, the virulence and pathogenicity of both isolates should be characterized.

## Supplementary material

10.1099/acmi.0.001097.v3 Uncited Supplementary Material 1.

## Abbreviations

blast: Basic Local Alignment Search Tool
CIs: confidence intervals
CMC: carboxy methyl cellulose
CNF: Cellulosic Nano-fibrils
CNF: Cellulosic Nano-fibrils
CR: Congo Red
CrI: cellulose crystallinity index
CS: crystalline size
CU: colour units
FTIR: Fourier transform infrared spectroscopy
G: guaiacyl
HBI: hydrogen-bonding index
LB: Luria–Bertani
LOI: lateral order index
MSM-L: minimal salt media supplemented with lignin sulphonate
NCBI: National Center for Biotechnology Information
SEM: scanning electron microscopy
SLC: surface lignin coverage
TCI: total crystallinity index
TSA: tryptic soy agar
XPS: X-ray photoelectron spectroscopy
XRD: X-ray diffraction
